# A meta-analysis of environmental sequencing data reveals the global distribution and hidden diversity of marine anaerobic ciliates

**DOI:** 10.1093/ismeco/ycag306

**Published:** 2026-10-21

**Authors:** Anna Schrecengost, Erin Frates, Alia Al-Haj, Robinson W Fulweiler, Roxanne A Beinart

**Affiliations:** Graduate School of Oceanography, University of Rhode Island, 215 South Ferry Rd, Narragansett, RI 02882, United States; Graduate School of Oceanography, University of Rhode Island, 215 South Ferry Rd, Narragansett, RI 02882, United States; Department of Biology, Boston University, 5 Cummington Mall, Boston, MA 02215, United States; Department of Earth and Environment, Boston University, 685 Commonwealth Ave, Boston, MA 02215, United States; Department of Biology, Boston University, 5 Cummington Mall, Boston, MA 02215, United States; Department of Earth and Environment, Boston University, 685 Commonwealth Ave, Boston, MA 02215, United States; Graduate School of Oceanography, University of Rhode Island, 215 South Ferry Rd, Narragansett, RI 02882, United States

**Keywords:** anaerobic protists, marine anoxia, meta-analysis, phylogenetic placement, protist metabarcoding

## Abstract

Anaerobic protists are diverse, ecologically important members of oxygen-depleted microbial communities, acting as grazers, nutrient cyclers, and partners in multi-domain associations, yet remain understudied relative to anaerobic prokaryotes. Ciliates are particularly abundant and diverse in these habitats, but their global diversity and distribution are largely unknown. Here, we conducted a meta-analysis of public 18S rDNA datasets, along with one dataset generated here, to assess the global diversity and ecology of marine anaerobic ciliates. Using a novel pipeline, we processed 2854 samples from 42 studies spanning 19 habitat types. We recovered 3196 anaerobic ciliate amplicon sequence variants (ASVs) across all described lineages. Based on clade-specific divergence thresholds derived from phylogenetic distances, 28.4%–46.3% of ASVs qualified as novel. Most sequences belonged to a poorly described plagiopylean clade, suggesting a large reservoir of undescribed diversity in this clade. Community comparisons revealed close phylogenetic similarities between some shallow-water and deep-sea assemblages, suggesting that shared redox conditions may shape communities more than water depth. Our results demonstrate that marine anaerobic ciliates are globally distributed, taxonomically diverse, and rich in novel lineages. This study provides a framework for leveraging environmental sequencing data to better understand the diversity and ecology of neglected protist lineages and under-sampled habitats.

## Introduction

Protists are among the most common and abundant organisms on the planet—some estimate that they may constitute over 85% of the overall eukaryotic diversity in the sunlit ocean, with similarly high proportions likely across other environments [1]. These single-celled eukaryotes play essential ecological roles, particularly in aquatic environments: they constitute a major proportion of microbial activity and biomass, cycle essential nutrients, and are major contributors to the biological carbon pump, which sequesters ~2.5 Pg carbon per year [2–6]. Most, if not all, of these processes are involved in the delicate biogeochemical feedback loops that regulate our rapidly changing climate, making it urgent to characterize this “unseen majority.” Specifically, by understanding the diversity of anaerobic protists and the factors that influence their community composition, we can begin to understand how they will affect and be affected by climate change [7]. Advancements in DNA sequencing and metabarcoding have greatly expanded our ability to study previously elusive protistan species and allowed us to explore protistan communities from previously inaccessible or largely unexplored environments, including oxygen-depleted marine environments [8, 9]. Most metabarcoding studies of marine protists are small-scale and limited in geographic scope, capturing a diversity of habitats but often employing different methods to do so, which complicates our ability to coherently synthesize this information. Extensive global sampling efforts focused on planktonic species [1, 10, 11], with relatively few targeting benthic or anaerobic taxa [5].

While most eukaryotic life relies on oxygen for respiration, a huge diversity of protists live in anoxia, and ciliates are particularly active, large, and diverse members of these understudied communities. They are a diverse and versatile group of protists known for their ability to establish various symbiotic partnerships [12–14] and inhabit a wide array of environments, including oxygen-depleted habitats such as aquatic sediments, the hypoxic water column, and the digestive tracts of animals [15–19]. Members of Ciliophora have transitioned from an aerobic to anaerobic lifestyle multiple times throughout their evolution [15, 20, 21], and representatives adapted to some degree of oxygen depletion, ranging from microaerophilic to obligately anaerobic [22, 23], are found across virtually all ciliate classes. Whereas anaerobic prokaryotes are relatively well-studied and appreciated for their large impact on biogeochemical cycles and the production of greenhouse gases [24, 25], microbial eukaryotes have been largely neglected in our accounting of anaerobic microbial communities. Anaerobic ciliates originating from marine environments have been especially overlooked, as most described species originate from freshwater habitats [22]. As hypoxic zones of the ocean have been expanding and the concentration of dissolved oxygen in aquatic habitats has been continuously decreasing over the past several decades [26, 27] a full accounting of marine microbial communities grows in importance.

Given that much of our current understanding of ciliate diversity stems from morphological surveys without any known DNA sequences [28], metabarcoding is a promising approach to uncover novel lineages and assess the full diversity and distribution of ciliate groups. Culture-based [12, 15, 16, 22, 29–34] and molecular studies [17, 35–42] of oxygen-depleted marine environments have been crucial in our understanding of the genetic diversity of anaerobic ciliates, generating most of the sequences on which our reference phylogenies and taxonomic databases are based and uncovering novel lineages, some of which are thus far understood to be endemic to specific locations [41]. Various geographically limited metabarcoding studies targeting protists from anoxic environments [42–44] have used older pyrosequencing technologies. Newer, Illumina-based environmental sequencing studies largely target marine habitats which are not explicitly anoxic, but which contain samples that are likely at least partially oxygen-depleted, such as intertidal [45–47], hydrothermal vent [48, 49], methane seep [50], and deep sea [51] environments, and of protists in bottom waters adjacent to hydrothermal vents [52, 53]. Many of these studies reported anaerobic ciliate taxa. However, thus far studies targeting microbial eukaryotes living in oxygen-depleted environments have been localized, and no efforts have been made to synthesize this wealth of sequencing data in a meta-analysis.

A comprehensive examination of existing marine anaerobic ciliate sequencing data would allow us to gain a better understanding of their global distribution and overall diversity (our current knowledge of which is primarily limited to local, culture-based studies) as well as assess the potential for novel lineages. Previously, we used culture–based approaches to survey marine anaerobic ciliates from intertidal sediments in Southern New England, and recovered a diversity of *Plagiopyla* and *Metopus* species, some of which were novel [29, 54, 55]. Here, we expand on this by mining publicly available marine 18S rDNA datasets for anaerobic ciliate sequences using a novel, publicly available Snakemake pipeline which employs phylogenetic placement to identify and assign denoised sequences to known anaerobic lineages. Phylogenetic placement can outperform traditional pairwise alignment-based taxonomic assignments [56] and is useful particularly for underrepresented groups because it incorporates phylogenetic information and retains deeply divergent sequences. Given how frequently novel lineages of anaerobic ciliates are discovered with low-throughput methods [15, 31, 41], we hypothesize that divergent sequences are likely abundant in under-explored anoxic environments.

We focused our analysis on three ciliate lineages in which anaerobiosis is a universal trait: the APM clade [15], Plagiopylea [30], and Anaerocyclidiidae [31]. It is thought that these lineages underwent independent transitions to anaerobiosis, and, therefore, any sequences which are assigned to them are most likely anaerobic. Additionally, these are the most commonly recovered anaerobic lineages in both culture-based and independent studies (Table S1). Although molecular data of anaerobes outside of these lineages exist, e.g. of isolated genera within Prostomatea and Litostomatea [57, 58], they are sparse, and these Classes consist primarily of aerobic or microaerophilic species. It is unlikely that phylogenetic placement at the resolution provided by short 18S amplicons would reliably distinguish anaerobic members within these groups. A comprehensive survey of anaerobic ciliate diversity across all ciliate classes would require different methods which are beyond the scope of this study. Here, we use the term “anaerobic ciliates” to refer operationally to ciliate amplicon sequence variants (ASVs) that are phylogenetically assigned to the APM clade, Plagiopylea, or Anaerocyclidiidae.

The goal of this study was to use publicly available data to explore anaerobic ciliate diversity across as many marine oxygen-depleted habitats as possible in order to explore their total global diversity, distribution across depths and among habitat types, and identify potential novel lineages. Our results have implications not only for our understanding of anaerobic ciliate diversity but also provide us with more information about which habitats and groups should be prioritized for more robust sampling and illustrate the power of a meta-analysis approach for understudied and -sampled protist groups. This approach holds promise for advancing our understanding of neglected microbial eukaryotes, particularly those from extreme, inaccessible habitats, by leveraging the wealth of existing sequencing data deposited in public repositories, and the pipeline presented here provides a framework for other researchers to do so.

## Materials and methods

### SRA to ASV Snakemake pipeline: raw data retrieval, processing into per-sample ASVs, and taxonomic assignment

We performed a literature review, ending in January 2024, to identify 18S rDNA high-throughput Illumina sequencing datasets that originated or possibly originated from oxygen-depleted habitats, either from habitats known to be oxygen depleted [59] or that reported the presence of taxa from ciliate lineages known to be exclusively anaerobic. Oxygen concentration data were not available for virtually any of the samples. Additionally, we included one new study of sediment 18S rRNA gene sequences that we generated from Narragansett Bay, Rhode Island (USA). All studies included in our analysis are listed and described in Table S2 and more information about data generation, study selection, and metadata is available in the Supplemental Methods.

Raw data retrieval and processing into ASVs was carried out via QIIME2 and Snakemake (Fig. S1). All steps were implemented as modular Snakemake rules and executed in a reproducible environment managed by Conda. Primer removal, merging of paired end reads, and denoising were performed in QIIME2 using cutadapt, vsearch, and deblur, respectively [60–63]. Taxonomic assignment of the resulting representative sequences was performed using a Naive Bayes classifier trained on the PR^2^ reference database [64]. Classification output was used for downstream filtering, resulting in per-study count tables for sequences assigned to Ciliophora as well as Unassigned sequences. After all studies were run through the Snakemake pipeline, Ciliophora-assigned and Unassigned ASVs and ASV count tables, respectively, were merged for downstream processing.

### Multi-level phylogenetic placement of ASVs onto reference eukaryotic, ciliate, and anaerobic ciliate clade phylogenetic trees

The Ciliophora-assigned and unassigned ASVs were phylogenetically placed onto a series of reference trees to identify and extract anaerobic ciliate sequences (ASVs ranged in size from 119–325 bp). Previously published eukaryotic and ciliate reference trees, which were designed for the phylogenetic placement of short reads, were used for this purpose [65, 66]. Anaerobic ciliate clade trees were constructed for the placement of per-clade ASVs (details available in the Supplemental Methods; Figs S2–S4; Files S1–S6). For the phylogenetic placements, we followed a multi-level phylogenetic placement strategy as described elsewhere [65, 67]. ASVs that were unassigned by QIIME2 taxonomic assignment were phylogenetically placed onto the Eukaryota reference tree. Ciliate sequences were extracted from those placements, combined with the sequences assigned to Ciliophora with QIIME2, and then placed onto the Ciliophora reference tree. Sequences assigned to each anaerobic ciliate clade were extracted from these Ciliophora placements and placed onto their respective reference trees. In the case of the APM clade, sequences that were assigned to Armophorea on the Ciliophora tree included all APM lineages, following the taxonomic scheme described in Ratjer and Dunthorn [66].

Phylogenetic placement was conducted as follows: ASVs were aligned to the full-length reference multiple sequence alignment using PaPaRa v2.5 [68], which performs phylogeny-aware alignment extension with the reference phylogenetic tree, reference alignment, and query sequences (ASVs) as input. This alignment was split into reference and query subsets with the epa-ng --split command [69]. Maximum likelihood model evaluation for the reference alignment was performed using the raxml-ng --evaluate command, which identifies the best-fitting model parameters for placement [70]. Phylogenetic placement of query sequences onto the reference tree was conducted using EPA-NG, with filtering parameters to retain the top 100 placements per query with an accumulated likelihood weight ratio (LWR) of 0.99. This produced a .jplace file containing the distribution of placements for each query across the reference tree.

### Analysis of phylogenetic placement results and anaerobic ciliate sequences

Gappa [71] was used to analyze and visualize phylogenetic placement results. High-confidence phylogenetic placements of anaerobic ciliate ASVs were filtered based on placement uncertainty (EDPL ≤0.05). Taxonomic assignments were generated from these placements using the command gappa examine assign.

For each reference tree, phylogenetic placements were visualized with the Interactive Tree of Life (iTOL v7) [72]. The global distribution of sampling locations was visualized by plotting the geographic coordinates from sample metadata using the sf and ggplot2 packages in R. ASV relative abundance as well as agglomerated taxonomic presence/absence were calculated per sample, and fractional taxonomic abundance plots were created with the PHYLOSEQ package in R [73]. Low abundance reads (<2) for each sample were removed prior to analysis.

The phylogenetic divergence between anaerobic ciliate communities from different habitat types was assessed using both the Kantorovich-Rubinstein Distance (KRD) and squash clustering. Prior to analysis, .jplace files were split per sample and grouped by habitat type. The KRD is a generalization of Unifrac distance, which calculates the minimum effort needed to transform one community’s placement distribution into another’s by moving mass along the tree [74]. Squash clustering is a hierarchical clustering method designed for phylogenetic placement data [75].

For each anaerobic ciliate clade, per-clade phylogenetic trees were re-inferred by re-aligning clade-assigned ASVs with reference sequences. Following tree inference, we assessed the phylogenetic distances between ASVs and references by computing the minimum patristic distance between each query and the reference sequences, yielding a per-query estimate of evolutionary divergence from the closest known relative. These distances were visualized using ridge plots in R to compare clade-specific patterns of phylogenetic novelty. Percent identity to the closest reference sequence for each query was also determined via BLASTn against the PR^2^ database.

ASVs were categorized as “novel” sequences based on sequence divergence as calculated via patristic distances. For each anaerobic ciliate clade, the average and maximum patristic distance between reference sequences were calculated, and these were used as thresholds. These thresholds range from 0.115–0.286 for the mean patristic distance and 0.203–0.469 for the maximum (Table S3). For each clade, ASVs that had greater patristic distances to their closest neighbor in the reference tree than these thresholds were classified as novel (Table S3).

## Results and discussion

### Recovery of a diversity of free-living anaerobic ciliate sequences from global marine environmental datasets

In total, we processed 2854 samples from 43 studies (Fig. 1, Table S4) and recovered 36 358 ciliate-assigned ASVs in 2030 samples and 3196 anaerobic ciliate ASVs across 649 samples. Of those anaerobic ciliate ASVs, 680 were originally unassigned at the phylum or higher taxonomic level and then identified with our phylogenetic placement pipeline (Fig. S5). Anaerobic ciliates were recovered from all habitats surveyed except mangrove sediment and bathypelagic water (Fig. 1). We found anaerobic ciliates from every free-living marine genus that we surveyed in this study, as well as many sequences unrelated to known genera. Additionally, we recovered ciliate sequences from across virtually the entire diversity of Ciliophora, with particularly dense placements on the genera *Discotricha*, *Lopezoterenia*, and *Leegaardiella*, as well as an early-diverging lineage within Scuticociliatia (Fig. 2). Although we do not investigate ciliate sequences outside of the anaerobic lineages surveyed here, the recovery of these sequences may reflect both the remarkable ecological flexibility of ciliates and the heterogeneous nature of oxygen depletion in marine habitats, which can exist in microniches both spatially and temporally, e.g. in dynamically mixing hydrothermal vent fluid or patchiness associated with surfaces, and in sediment [19, 76, 77]. Assuming that these sequences originated from live ciliates, it is possible that the samples included in this analysis are mismatched with the microniches where anoxia generally exists, and that many, if not the majority, of samples included originated from primarily oxic or micro-oxic habitats. Of course, it is also possible that some of the recovered sequences represent uncharacterized anaerobic lineages, or anaerobic lineages which were not included in this analysis (e.g. *Legendrea* and *Dactylochlamys* in the Litostomatea [57, 78], potential anaerobic *Euplotes* (Spirotrichea), [79], *Copemetopus* [80], and *Holophrya ovum* and *Apolagynus* in the Prostomatea [58]).

**Figure 1:**
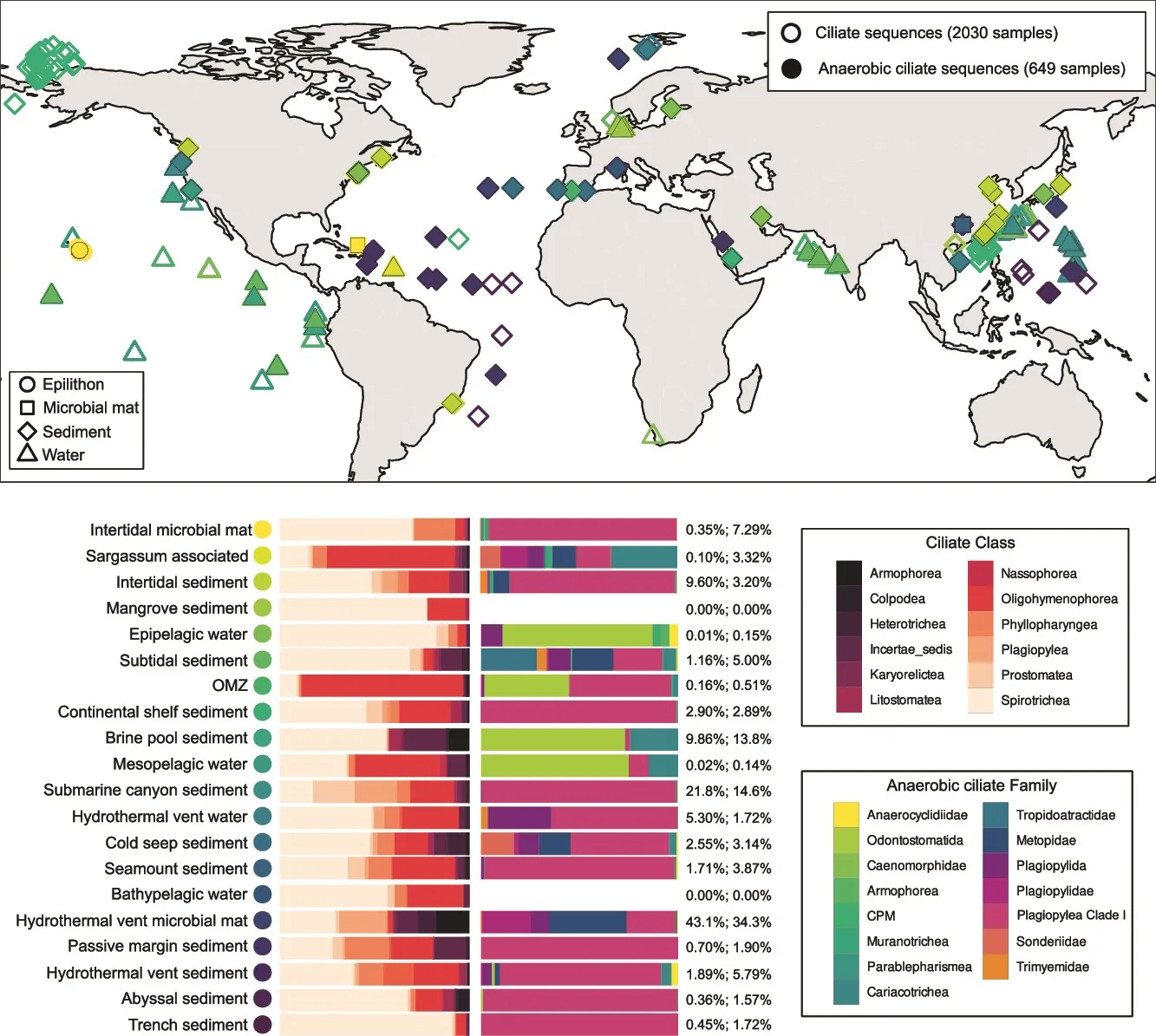
Top: map showing the location of samples included in this study that contained ciliate sequences (empty outlined points) and anaerobic ciliate sequences (filled-in points). Points are colored by habitat type, which is described in the legend at the bottom, and substrate type, which is described in the legend within the map. Bottom: compositional bar plots describing the relative abundance of different ciliate classes (left) and anaerobic ciliate families (right) recovered in the samples, separated by habitat type. To the right of compositional bar plots for each habitat type are the percent of total ciliate reads that were assigned to anaerobic clades (left) and proportion of total ciliate ASVs that were anaerobic clades (right).

**Figure 2:**
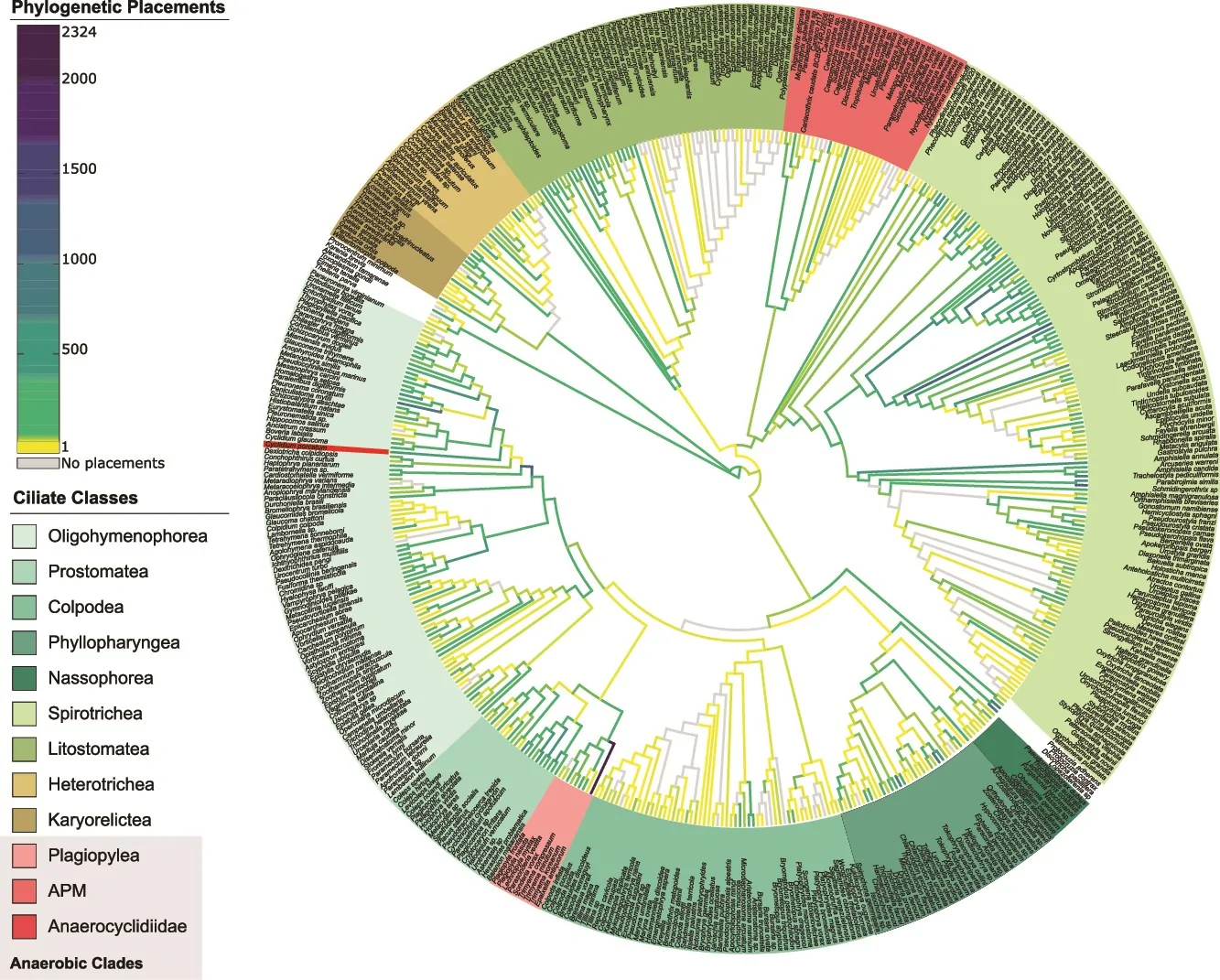
Phylogenetic placement of pre-filtered ASVs onto Ciliophora reference tree. A full-length 18S rRNA gene reference phylogenetic tree [66] was used in the phylogenetic placement of pre-filtered ASVs, which were either assigned to Ciliophora in QIIME2 or were unassigned in QIIME2 but later assigned to Ciliophora via phylogenetic placement onto a reference Eukaryota phylogenetic tree. Ciliate classes are colored at the tips, and anaerobic clades are highlighted. Color on branches indicates the distribution of top phylogenetic placements for each query, which corresponds to their phylogenetic placement-based taxonomic assignment. The Ciliophora reference tree was rooted with representative sequences from the sister alveolate phyla Dinoflagellata and Apicomplexa.

While we did recover sequences spanning the full diversity of the anaerobic ciliate groups included in our analysis, some were represented more than others. In particular, the relative paucity of odontostomatid sequences most likely reflects primer biases rather than true absence, as most “universal” primers fail to amplify their divergent 18S rRNA gene sequences, including those used in the studies compiled here (Table S2). A recent analysis by Mendez-Sanchez *et al.* [33] recovered an abundance of novel odontostomatid sequences from environmental datasets, reflecting their unexpected diversity and underrepresentation in environmental analyses.

The only habitats in which we recovered ciliate reads, but no anaerobic ciliate reads, were mangrove sediment and bathypelagic water. In mangrove habitats, this likely reflects undersampling, as relatively few samples (*n* = 20) and ciliate reads (104 ASVs) were recovered, despite evidence that mangrove sediments harbor anaerobic ciliates [54, 81]. In general, global environmental sequencing data from mangroves and tropical estuaries is lacking [82]. Additionally, the compositional nature inherent to metabarcoding data complicates interpretation of zero values, particularly in a meta-analysis such as this one where the biases inherent in microbiome data analysis are multiplied [83]. Also, protist spatial and temporal distribution is heterogeneous, and anaerobic protists are traditionally thought to be rare, although it is likely they are not as rare as once thought [9, 78]. The bioinformatic methodology in this study was designed to minimize differences between datasets—when possible, we used consistent parameters and trimmed reads to identical regions. Additionally, we used the conservative deblur algorithm for denoising, which was designed for use in meta-analyses [61]. We also used a phylogenetic placement method, which allowed us to analyze all ASVs together regardless of primer set and place the sequences into a phylogenetic context [84].

### Anaerobic ciliate communities appear similar across some deep sea and shallow water habitats

To compare anaerobic ciliate communities across different global marine habitat types, we performed phylogenetic placement-based beta diversity analyses (Figs 3 and S6). We observed that water column samples from oxygen-minimum zones, anoxic basins, estuarine water, etc. are more similar to each other than to other samples. We also observed close similarity between anaerobic ciliate communities from hydrothermal vents, intertidal zones, and cold seep sediments. Microbial mat and sediment samples from hydrothermal and intertidal or subtidal environments consistently grouped together. Specifically, hydrothermal vent microbial mat and subtidal sediment, hydrothermal vent sediment and intertidal sediment, and deep cold seep sediment and intertidal microbial mat were most similar, respectively.

**Figure 3:**
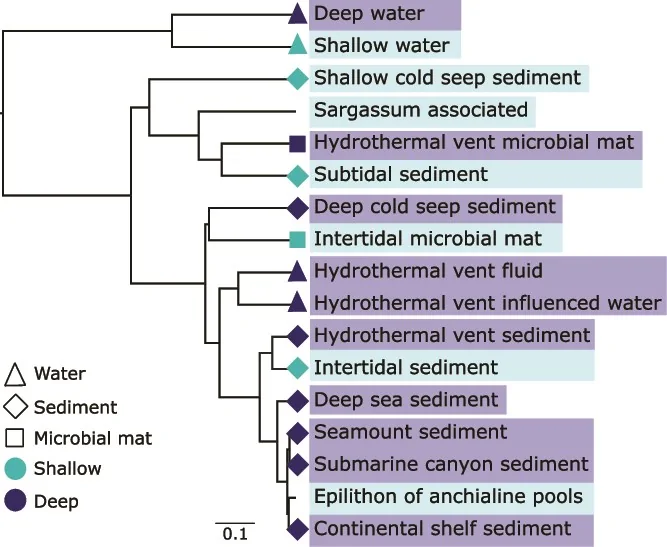
Hierarchical clustering of KRD between samples grouped by habitat type. Branch tips are colored by water depth, with deep defined as greater than 300 m, and branch node shapes indicate sample substrate. KRD is a generalization of the weighted Unifrac beta diversity measure.

Presence/absence analyses supported these patterns, suggesting that deep-sea cold seep and hydrothermal vent habitats shared similar anaerobic ciliate assemblages with intertidal and subtidal habitats (Fig. 4). Most habitats were dominated by the presence of Plagioplea Clade I, with Plagiopylida, Cariacotrichea, and Anaerocyclidiidae also very prevalent across many habitats. Sonderiidae ciliates were only recovered from intertidal, subtidal and cold seep sediment, as well as hydrothermal vent microbial mat and Sargassum-associated samples, while water column habitats contained fewer detectable anaerobic ciliate taxa than sediment and microbial mat habitats.

**Figure 4:**
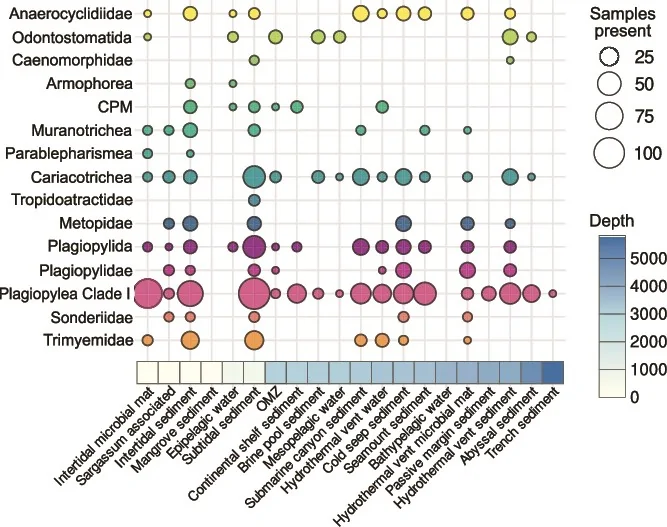
Presence-absence bubble plot illustrating prevalence of major anaerobic ciliate taxa across marine oxygen-depleted habitats. Habitat types are organized and colored by average depth. Bubble sizes correspond to the number of samples in which the taxa were present for each habitat.

These clustering patterns suggest that similarities in anaerobic ciliate community composition across deep-sea and shallow-water habitats may reflect shared biogeochemical conditions rather than the influence of depth/pressure. Of course, one limitation of synthesizing published data is a lack of standardized metadata; here, we lack information on the biogeochemistry of these samples, including oxygen concentrations, but we do have knowledge of the biogeochemistry that is typical of these habitat types. Hydrothermal vent fluid samples clustered with sediment and microbial mat samples, while other water samples (e.g. OMZ) did not. This may be due to the chemical similarity of vent fluid and sediment - vent fluids are enriched in reduced chemicals, such as hydrogen sulfide, methane, and hydrogen [39]; similarly, sediments contain redox gradients where these same chemicals accumulate as products of and fuel for anaerobic metabolisms [23]. In contrast, oxygen-deficient water columns such as OMZs are often not fully anoxic and generally lack appreciable concentrations of sulfides or other chemical reductants [85]. Additionally, the clustering observed between shallow-water microbial mats and deep-sea sediments (e.g. at cold seeps and hydrothermal vents, Figs. 3 and S5) may be influenced by shared physical structure, organic matter availability, and vertical redox gradients [86].

Anaerobic ciliates rely on the syntrophic consumption of e.g. hydrogen by their methanogenic, sulfate-reducing, or other symbionts to persist in anoxia [12, 22] and are important grazers in these habitats [87]. Therefore, the redox landscape is relevant to their community composition by enabling the microbial processes that sustain these symbioses, and by influencing the composition of free-living bacterial and archaeal communities. It is possible that the free-living bacterial and archaeal communities are similar across some of these habitats and that the similarities in anaerobic ciliate community composition are influenced by the interdomain biological interactions that occur between these groups. Beggiatoa microbial mats, for example, occur globally across shallow and deep-sea environments where oxic and sulfidic zones interface [88].

Interestingly, multiple previous studies have demonstrated that protist communities are significantly different across depth in the marine water column [89, 90] and across overlying water column depth in the sediment [91, 92]. Depth has been shown to greatly influence the composition of benthic ciliate communities [51] but not always pelagic communities [93]. In both deep-sea and shallow habitats, benthic protists have been found to be shaped more by depth than geography, where even geographically disparate communities are highly similar at the same depth profile [92, 94]. In the aforementioned studies, depth is often a proxy for many covarying environmental parameters: grain size [51, 92], dissolved oxygen content and surface productivity of the overlying water column [89, 90, 92–94], temperature, salinity [94], and dispersal limitation caused by bathymetry [51, 91] were found to exert the greatest influence over benthic protist communities, while oxygen and chlorophyll content [89, 90, 93] are important for the plankton. Therefore, in many cases, depth-driven differences in community composition may partially reflect oxygen gradients rather than depth or pressure.

The anaerobic ciliate communities that we recovered appear to follow the same principle. We found that the communities in some shallow habitats (e.g. intertidal sediments) are very similar to those in some deep-sea habitats (e.g. hydrothermal vent sediment), not because depth is irrelevant, but likely because these habitats share the environmental conditions that depth acts as a proxy for elsewhere. Oxygen-depleted sediments and microbial mat habitats have similar redox conditions, no light availability, high microbial biomass and organic matter loading, and are rich in reduced compounds such as hydrogen sulfide. Our results suggest that these shared conditions are what influence anaerobic ciliate community composition rather than depth or geographic distance. Indeed, identical anaerobic ciliate species have been isolated from similar habitats across the globe, and, in some cases, they even host identical archaeal symbionts: for example, a *Metopus* species isolated from intertidal habitats in the USA and Portugal was found to harbor *Methanocorpusculum* symbionts with identical 16S rDNA and ITS sequences [48, 49]. While the Baas Becking hypothesis, i.e. “everything is everywhere, but the environment selects,” has been largely disproven for biogeographically structured protist communities [9], it is possible that this hypothesis more accurately describes anaerobic protists, which have independently evolved the ability to exploit anoxic niches multiple times throughout their evolutionary history [22, 23]. These repeated transitions to anaerobiosis across multiple lineages involved the reductive evolution of mitochondrion-related organelles and the acquisition of syntrophic symbionts [15, 21], which may constrain anaerobic ciliates to similar ecological niches defined by redox chemistry and symbiont availability, rather than by geography or depth. Large-scale meta-analyses such as this one help us better understand the global diversity and distribution of different protist groups and how they are influenced by various environmental conditions. However, it is also important to consider that marker genes such as the 18S rRNA gene do not have enough phylogenetic resolution to reliably distinguish species, and so sequences which seem identical may be phylogenetically, pheno, or genotypically distinct.

### Recovery of a high prevalence of anaerobic ciliate sequences from undescribed plagiopylean clade and other divergent sequences

One of the most striking results from our analysis is the prevalence of Plagiopylea clade I ciliates across virtually all marine oxygen-depleted habitats surveyed (Figs 2 and 4). Not only was this the most common anaerobic ciliate taxa observed across all the samples (2206 out of 3196 (69%) of all anaerobic ciliate placements were on this clade), but even among all ciliate phylogenetic placements, by far the greatest density of placements was observed on this little-known clade (Fig. 2). Additionally, ciliates from this clade were found in habitats where no other anaerobes were recovered, such as some deep-sea sediments (Fig. 4). Taking a closer look at the Plagiopylea placement results (Fig. S2), most of these sequences were placed on the basal branch of this unidentified clade or on/near the “Epalxella antiquorum” branch.

Plagiopylea clade I represents a basal lineage within the Plagiopylea and contains representatives originating from a range of habitats: cold seep sediment, the Cariaco basin, the Black Sea, a benthic diatom film, sewage treatment sludge, an anoxic fjord, as well as a freshwater lake (Fig. S2). The freshwater lake representative harbors unique bacterial symbionts which generate ATP for the host via denitrification [95]. Combined with the described methanogenic archaeal symbionts of other plagiopylean ciliates and the huge diversity of habitats where it is found globally, this suggests a remarkable ecological diversity within this little-known anaerobic ciliate family. The abundance of ASVs assigned to this ill-defined group as well as its prevalence across the global habitats surveyed here suggests that there is a large pool of uncharacterized diversity of ciliates from this family, and future studies should prioritize understanding anaerobic protists and underexplored protist groups in general.

Approximately half of the ASVs assigned to the APM clade (203 out of 291) belonged to a poorly described subgroup within the APM, which we refer to here as the CPM clade (Cariacotrichea, Parablepharismea, and Muranotrichea; Fig. S3). All of these ciliate classes are little known, deep-branching, and have only been described a few times in the literature. There is currently only one known species from the class Cariacotrichea, *Cariacothrix caudata*, which has been isolated from the water column of the deep anoxic Cariaco basin water column [41]. Other sequences that are included in this clade in this and other analyses [96] were isolated from a deep-sea methane seep [50] and in an oxygen minimum zone [85]. After Plagiopylea Clade I and Plagiopylida ciliates, Cariacotrichea was the most prevalent group in our analysis, recovered from both shallow water and deep-sea habitats (Fig. 4). Parablepharismea contains two recently sequenced species isolated from brackish lagoon sediment [32], as well as species from salt pond sediment, although those are not included here because they are too similar to the other species [15]. Muranotrichea was only recently established as a novel ciliate class and contains three described genera which were isolated from shallow freshwater, marine, and brackish sediment, [15, 97], as well as from the deep sea [34].

Many of the sequences that we recovered were highly divergent from known references, based on both pairwise nucleotide identity and phylogenetic (patristic) distance (Fig. 5, Table S3, Fig. S7). Sequences assigned to the majority of groups surveyed here showed broad and diffuse distributions of pairwise identity with reference sequences. Many sequences fell near 90% identity, the approximate threshold for sequences within the same family [65]. Sequences assigned to Odontostomatida were the most divergent in terms of phylogenetic distance, although these large distances are likely unreliable due to this group’s poorly resolved phylogenetic position and long-branching nature in Ciliophora phylogenies [58]. Recovered sequences from Plagiopylea Clade I had an average phylogenetic distance of 0.27 ± 0.14 s.d.

**Figure 5:**
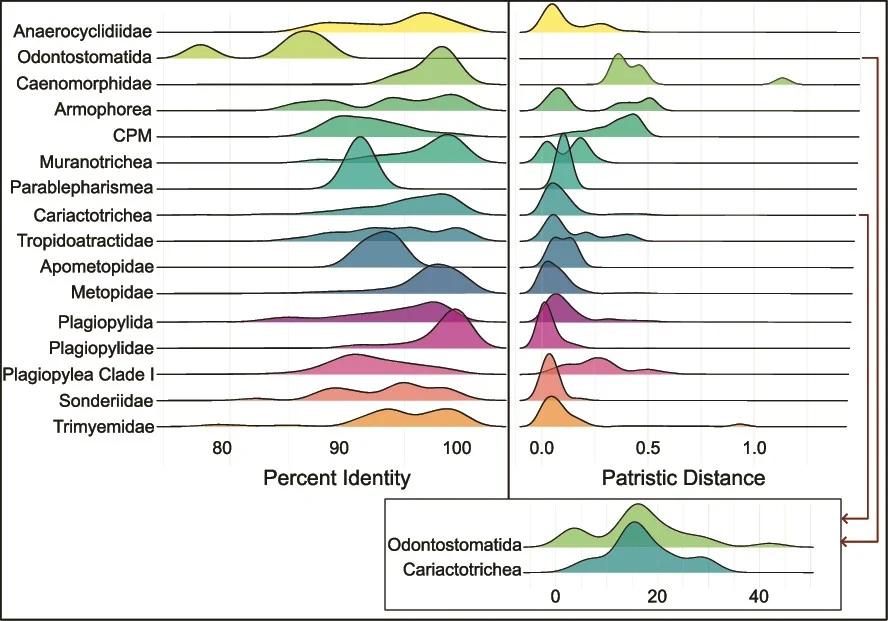
Left: pairwise percent identity of anaerobic ciliate sequences to their closest reference hit in the PR^2^ database, as determined by BLASTn. Sequences are grouped by taxa and results are shown in a ridge plot. Right: Patristic distance of anaerobic ciliate sequences to the sequence to which they are most closely related phylogenetically, based on a re-inferred phylogenetic tree which includes both reference and ASV sequences. Sequences are grouped by taxa and results are shown in a ridge plot. The patristic distance refers to the sum of the branch lengths between the ASV and the closest reference sequence. Distances larger than 1.5 are excluded from the main plot and instead shown in the inset at the bottom.

We classified ASVs as novel if the phylogenetic distance to their closest neighbor on the reference tree (which was calculated from a phylogenetic tree containing both ASVs and reference sequences) was greater than the average phylogenetic distance among sequences within their assigned clades (Table S3). We also classified ASVs much more conservatively using the maximum per-clade phylogenetic distances as the threshold, and here we report the estimated proportion of novel ASVs per clade as a range. Averaged across all clades, the less conservative threshold was a patristic distance of 0.162 (± 0.085 s.d.), while the more conservative threshold was 0.30 (± 0.14 s.d.). Using these per-clade criteria, 31.8%–49.6% of the ASVs in Plagiopylea were novel, as were 8.9%–32.3% of the ASVs within the APM clade and 0%–14.78% of the ASVs in Anaerocyclidiidae. Smaller clades within these broader lineages contained various proportions of novel sequences. For example, 40%–57.6% of ASVs classified within Plagiopylidae Clade I were novel, whereas only 6.7% of ASVs from *Plagiopyla* were novel. We also recovered a large proportion of novel sequences assigned to the CPM clade (0%–61.5%). Overall, most of the anaerobic ciliate clades recovered in this study contained substantial proportions of novel ASVs (at least 20%).

A large-scale, global analysis such as this one allows us to explore which habitat types contribute the most novel anaerobic ciliate phylogenetic diversity, making them interesting targets for future exploration, sampling, and sequencing efforts. We can assess the phylogenetic novelty of sequences from different habitats and clades using our calculated phylogenetic distances as a proxy for divergence from described diversity. We found that hydrothermal vent, continental shelf, submarine canyon, and seamount sediment habitats had the highest average patristic distances (excluding habitats with fewer than 20 representative samples; Fig. S7), suggesting they harbor sequences that are more divergent from known anaerobic ciliate diversity and represent promising habitats for future exploration.

Additionally, we performed a rarefaction analysis of cumulative ASV discovery as a function of sampling effort for a subset of the data (ASVs which were generated with the Stoeck *et al.* [42] primer set: 1251 anaerobic ciliate ASVs across 901 samples and 22 studies) (Fig. S8). This revealed decelerating but unsaturated discovery across all groups (except for the APM clade, which increased linearly with sampling effort), suggesting that the broad habitat distributions and high proportions of novel sequences observed for the anaerobic ciliate clades surveyed here would not have been apparent from any single study, and that increased sampling would yield more diversity. Taken together, these results suggest that our meta-analysis uncovered a large amount of undescribed diversity which would not have been apparent in a smaller-scale analysis and demonstrates the utility of a phylogenetic placement-based meta-analysis approach such as this for the exploration of understudied protist groups and habitats.

## Conclusion

Anaerobic ciliates have been largely unaccounted for in our assessments of the diversity of anoxic microbial communities, despite their important ecological roles in biogeochemical cycling as grazers and via syntrophic associations with microbes from multiple domains of life. In this study, we leveraged publicly available 18S rDNA datasets to uncover a remarkable diversity of anaerobic ciliate sequences across geographically and physically disparate global marine habitats. The prevalence of divergent sequences, particularly within a poorly described plagiopylean clade (referred to here as Plagioypylea Clade I) and the deep-branching CPM clade, highlights the global ecological relevance and potential for discovery in these groups. Additionally, anaerobic ciliate community composition appeared not to be influenced greatly by water depth or geographic distance, potentially reflecting their extremophilic lifestyle and/or the multiple independent origins of anaerobiosis among ciliates, which could constrain these lineages to similar niches defined by redox chemistry. Of course, it is possible that this data cannot differentiate species- or strain-level diversity, and that these habitats share similar community profiles at some higher taxonomic level. Future work with higher resolution sequencing approaches like full-length rRNA gene amplicons or population genomics will help to resolve these types of questions.

For now, there is an abundance of publicly available short-read sequencing data from diverse habitats around the globe, which represent an underutilized resource in the study of poorly researched protist groups. Our publicly available pipeline provides a framework for other researchers to leverage these data for any protist lineage, given a reference phylogenetic tree. As environmental sequencing datasets continue to accumulate, meta-analyses such as this one will play an increasingly important role in the search for novel taxa, exploration of the diversity of little-known protist groups, and identification of the environments and taxonomic groups that should be prioritized for more intensive study via culture-based and ‘omics approaches, like metagenomics. Indeed, environmental sequences have already reshaped our understanding of the diversity and distribution of eukaryotic microbes in a wide variety of habitats; however, studies such as this one demonstrate that much remains to be uncovered, particularly in extreme and under-sampled habitats [1, 4, 9, 65, 98, 99].

## Supplementary Material

Supplementary_material_ycag306

## Data Availability

Raw 18S rDNA amplicon sequences which were generated in this study are available in the NCBI Sequence Read Archive (SRA) (Bioproject ID PRJNA1347111). The 18S rDNA sequence for *Sonderia* sp*.* PLNINIBRI3 is available on GenBank (accession number PZ305114). Scripts for bioinformatic analyses, including the Snakemake pipeline, are available on GitHub, https://github.com/aschrecengost/18S_meta-analysis/tree/main.
