# Supplementary material for: A meta-analysis of environmental sequencing data reveals the global distribution and hidden diversity of marine anaerobic ciliates
Source: ISME Commun. 2026 Oct 21;6(1):ycag306. doi: 10.1093/ismeco/ycag306 (PMC13738208; doi:10.1093/ismeco/ycag306)
Supplement: Supplementary file 1 [file supplementary_material_ycag306.zip › revised_SupplementalInformation_final.docx]

**Supplementary information for “A meta-analysis of environmental sequencing data reveals the global distribution and hidden diversity of marine anaerobic ciliates”**

Anna Schrecengost^1,a^, Erin Frates^1,2^, Alia Al-Haj^3,b^, Robinson W. Fulweiler^2,3^, Roxanne Beinart^1,a^

^1^University of Rhode Island, Graduate School of Oceanography, 215 South Ferry Rd, Narragansett, Rhode Island 02882, USA

^2^Boston University, Department of Biology, 5 Cummington Mall, Boston, MA 02215

^3^Boston University, Department of Earth and Environment, 685 Commonwealth Ave, Boston, MA 02215

^a^Corresponding authors: aschrecengost@uri.edu (AS); rbeinart@uri.edu (RB)

^b^Current affiliation: Smithsonian Environmental Research Center, 647 Contees Wharf Rd, Edgewater, MD 21037

**Contents**

**METHODS**

**REFERENCES**

**SUPPLEMENTAL TABLES**

**SUPPLEMENTAL FILES**

**SUPPLEMENTAL FIGURES**

**METHODS**

*Study selection and metadata*

We performed a literature review to identify 18S rRNA gene high-throughput sequencing datasets which might possibly contain marine anaerobic species and which were sequenced with Illumina technology. We searched Google Scholar with the following search terms: “marine sediment”, “intertidal”, "18S", "Illumina" “microeukaryote”, “ciliate”, “protist”, “anoxic”, “oxygen-depleted”, “hydrothermal vent”, “oxygen-minimum zone”, “cold seep”, “methane seep”. We also searched for 18S amplicon studies which reported the presence of anaerobic ciliate taxa. We only included peer-reviewed studies which were written in English. Additional studies were sometimes obtained through the papers which were referenced in the studies from our search results. This literature review was completed in January of 2024, and no studies published after that date were included in the analysis.

The study selection criteria included: those which conducted Illumina amplicon sequencing of any region of the 18S rRNA gene, deposited the raw .fastq files in some public repository that could be accessed (usually NCBI SRA; in some cases, the corresponding authors were contacted for these files), and those that included samples from marine habitats which were or could possibly be oxygen-depleted, either explicitly with O_2_ measurements, in habitats known to be oxygen depleted (1), or that reported the presence of anaerobic ciliate taxa.

Metadata was obtained from the NCBI SRA website for each study and occasionally supplemented from the primary literature if necessary. The metadata was curated to include following fields for all studies (Table S2): Sample ID, Study ID, latitude, longitude, depth, primer region, primer set, sequencing chemistry, env_1 (indicates whether the sample is marine or brackish), env_2 (indicates whether the sample originated from sediment, water, or microbial mat), env_3 (indicates the habitat type e.g. intertidal, subtidal, hydrothermal vent), env_4 (the sample description as provided from the original metadata file/the literature), env_5 (our own most detailed description of habitat). Other metadata fields were also included for some samples when relevant, e.g. oxygen concentration measurements from the TARA oceans dataset to identify anoxic samples.

*Sampling and sequencing of Narragansett Bay sediments*

Sediment samples were collected from three sites across Narragansett Bay, RI, USA: the Providence River Estuary, Wickford Harbor (Public Dock and Gardener’s Wharf) and Mid Bay (Table S2). Sediment cores (10 cm inner diameter and 30.5 cm long) were collected using either a 4.5 m long pole corer (Providence River Estuary and Wickford Harbor) or with SCUBA divers (Mid Bay). With each method the vertical structure of the sediment core as well as the flocculent surface layer were retained (2). These cores were subsampled into multiple depth horizons (1 cm deep, up to 6 cm). DNA from the top two horizons from all cores, as well as the additional four depth horizons from the Wickford Harbor sites, was extracted from the sediment samples using the Macherey-Nagel Nucleospin Soil Kit (Macherey-Nagel, Düren, Germany), following manufacturer’s instructions. 18S rDNA was amplified from this DNA using a universal primer set targeting the V9 region of the 18S rRNA gene: 1389F (5′-TTGTACACACCGCCC-3′) and 1510R (5′-CCTTCYGCAGGTTCACCTAC-3′) (3), as well as a Stramenopile-Alveolata-Rhizaria (SAR) specific primer set: SAR F (5'-AYTCAGGGAGGTAGTGACAAG-3') and SAR R (5'-RACTACGAGCTTTTTAACTGC-3') (4). The resulting amplicons were sequenced on an Illumina MiSeq at the RI Genomics and Sequencing Center to generate paired end reads (2x300 bp). The 18S rRNA gene sequence for *Sonderia* sp. PLNINIBRI3, which is used in the Plagiopylea reference tree, was originally generated in 2020 as described in (5), but not published at the time.

*SRA to ASV Snakemake pipeline: Raw data retrieval, processing into per-sample ASVs, and taxonomic assignment*

Raw data retrieval and processing into ASVs was carried out via QIIME2 and Snakemake, with all steps implemented as modular Snakemake rules (Figure S1). Parameters were kept as consistent as possible to facilitate combinations of ASV tables, and when possible reads were trimmed to the exact same primer region. Raw FASTQ files from each study were retrieved from the NCBI Sequence Read Archive (SRA) via user input of SRA accession ID into the Snakefile. Run metadata was obtained via the Entrez Direct utilities esearch and efetch and filtered to extract valid SRR sample identifiers (6). FASTQ files were downloaded using parallel-fastq-dump, which supports multithreaded retrieval and automatic splitting of paired-end reads.

Raw FASTQ files were renamed to QIIME2 convention and then imported into QIIME2. Adapters and primers were removed with qiime cutadapt trim-paired with user-defined primer sequences and read-through trimming where necessary (7,8). Forward and reverse reads were merged using qiime vsearch merge-pairs, allowing staggered alignment with user-defined thresholds for mismatch tolerance and minimum overlap length (9). Merged reads were quality filtered with qiime quality-filter q-score, and denoising was performed using Deblur inside QIIME2 with reads trimmed to a user-defined fixed length and denoised against the Protist Ribosomal Reference Database (PR^2^) provided in FASTA format (10,11). This process generated for each study a FASTA file of ASVs, an ASV count table, and accompanying deblur statistics. These statistics along with the raw reads, primer-trimmed reads, merged reads, and filtered reads were visualized with qiime demux summarize on view.qiime2.org to diagnose potential issues during sequence processing.

Taxonomic assignment of representative sequences was performed using a Naive Bayes classifier trained on the PR^2^ reference database, implemented via qiime feature-classifier classify-sklearn (12). Classification output was used for downstream filtering: sequences classified under the phylum Ciliophora were filtered from both the feature table and representative sequence set using qiime taxa filter-table and qiime taxa filter-seqs, respectively, and the same was done for sequences which were classified under Unassigned at the kingdom or phylum level.

*Generation of anaerobic ciliate clade phylogenetic trees*

Full length 18S rRNA gene datasets were manually curated based on the literature and dereplicated at 99% identity with the aim of including one sequence for each species. For Plagiopylea, we included both cultured and environmental strains which have been published in phylogenies previously and used Prostomatea as an outgroup (13,14) (Figure S2, Files S4-S6). For Anaerocyclididiiae, we used a sequence alignment from a recently published study which established this family as a novel anaerobic lineage (13) (Figure S4). For the APM clade, we included both cultured and environmental sequences which have been included in published phylogenies of this group and also supplemented with environmental sequences from PR^2^ which were assigned to Cariacotrichea (14,15) (Figure S3, Files S1-S3). Full length 18S rRNA sequences were obtained from GenBank. Per-clade alignments were generated using the MAFFT algorithm and the progressive method L-INS-I (15). Alignments were manually trimmed using AliView (16) and phylogenetic trees were generated using a Maximum likelihood method in RaxML under the GTR+GAMMA substitution model with 200 independent runs and 1000 bootstrap replicates (17). Files which list the GenBank IDs of reference sequences and describe the taxonomy of each branch in the phylogeny were generated as well. Files associated with the generation and use of the Plagiopylea and APM clade trees, including the reference alignments, rooted trees, and taxonomy are provided in the Supplement (Files S1-S6).

*Analysis of phylogenetic placement results and anaerobic ciliate sequences*

Gappa (18) was used to analyze and visualize phylogenetic placement results. The expected distance between placement locations (EDPL) and likelihood weight ratio (LWR) of queries and placements were calculated with gappa examine lwr-list and gappa examine edpl, respectively. Queries with EDPL > 0.05 were filtered out using a custom script and seqkit (19) and excluded from further analysis. In this way, sequences with diffuse and uncertain placements were removed, only retaining those placed with high confidence in a specific area of the tree. To translate phylogenetic placements into taxonomic annotations, we used the command gappa examine assign. This step maps the likelihood weight of each phylogenetic placement onto a user-specified taxonomy. The command was executed with the --best-hit strategy, which assigns each query to the taxon associated with its highest-likelihood placement, and a consensus threshold of 0.7, which sets the minimum proportion of descendant tips that must share a taxonomic rank for the query to be assigned to an internal node.

To assess phylogenetic divergence between anaerobic ciliate communities from different habitat types, we performed another phylogenetic placement of all anaerobic ciliate ASVs onto a modified Ciliophora reference tree, which was re-inferred after the addition of all of the sequences from the anaerobic clade reference tree. Prior to analysis, .jplace files were split per sample using gappa edit split and the associated ASV count table. Only samples containing a minimum number of placements were retained for downstream analysis (at least 5). We grouped per-sample .jplace files by habitat type and used the command gappa analyze krd to compute the Kantorovich-Rubinstein Distance (KRD) between these grouped samples. The KRD is a generalization of Unifrac distance which allows read placements anywhere in the tree and allows for uncertainty in placement. It calculates the minimum effort needed to transform one community’s placement distribution into another’s by moving mass along the tree (20). The KRD matrix was converted to a distance object in R and hierarchically clustered using the UPGMA (Unweighted Pair Group Method with Arithmetic Mean) algorithm via the hclust() function.

We also performed squash clustering on these same groups of samples, which is a hierarchical clustering method designed for phylogenetic placement data. Squash clustering reduces each sample's placements into a single averaged distribution in the tree, averages the placement profiles below each node in the tree, then measures the distance between these averaged distributions to define branch lengths (21). We applied squash clustering via the command gappa analyze squash to the same per-habitat .jplace files, and the tree topology was congruent with that which we obtained with KRD. Because uneven sampling depth and primer bias can compromise distance-based calculations, we were cautious about interpreting community divergence based on KRD alone. The observed clustering patterns from KRD were consistent with those obtained using squash clustering (Figure S5), which is more robust to differences in read depth because each sample is represented as a normalized placement distribution. The agreement between both methods suggests that our results reflect a biological signal rather than methodological bias.

For each anaerobic clade, we re-inferred phylogenetic trees by combining clade-assigned ASVs with reference sequences and aligning them using the MAFFT algorithm with the --addfragments option, which is meant to add short sequences to a multiple sequence alignment, and maintaining the original alignment length. Maximum likelihood tree inference was performed with multithreaded RAxML v8.2 under the GTR+GAMMA substitution model with 200 independent tree searches and 1000 bootstrap replicates. To preserve the reference phylogenetic tree topology, we used the per-clade reference trees as constraint trees with the -g parameter. Following tree inference, patristic distances, which is a type of phylogenetic distance defined as the sum of branch lengths connecting two nodes, were calculated between each query and reference sequence using the cophenetic.phylo() function from the ape package in R (22). For each query, the minimum patristic distance was extracted, yielding a per-query estimate of evolutionary divergence from the closest known relative. These distances were visualized using ridge plots in R to compare clade-specific patterns of phylogenetic novelty. Additionally, for each query sequence BLASTn searches were performed against the PR^2^ database to identify the percent identity of each ASV to its closest reference sequence (23). Percent identity values from the top BLAST hits were extracted and visualized using ridge plots in R.

As using the maximum reference patristic distance is the most conservative approach to categorize novel sequences, for each clade we provide a range of percentages of novel sequences, with the number calculated using the more conservative maximum threshold at the bottom of the range. Sequences which were assigned to Odontostomatida or whose closest neighbor was an odontostomatid were excluded from this classification, because the poorly resolved phylogenetic position of this group renders calculated phylogenetic distances unreliable (24).

We performed rarefaction analysis of cumulative ASV discovery as a function of sampling effort, restricting the analysis to ASVs originating from reads generated by the Stoeck et al. 2010 primer set, because this ensures comparability while representing the largest proportion of samples in our dataset (22 studies, 901 samples, 1251 anaerobic ciliate ASVs). ASVs were clustered at 98% and 99% sequence identity using Clusterize as implemented in the DECIPHER R package (25). For each group and clustering threshold, rarefaction analysis was performed using the specaccum() function in the vegan R package (26).

**REFERENCES**

1.	Fenchel T. and Finlay B. J. Ecology and evolution in anoxic Worlds. Oxford Series in Ecology and Evolution. ISBN 019-854838-9 (H.b.) and 019-854837-0 (P.b.). J Evol Biol. 1996;9(2):259–60.

2.	Fulweiler RW, Nixon SW. Net sediment N2 fluxes in a southern New England estuary: variations in space and time. Biogeochemistry. 2012 Nov;111(1–3):111–24.

3.	Amaral-Zettler LA, McCliment EA, Ducklow HW, Huse SM. A method for studying protistan diversity using massively parallel sequencing of V9 hypervariable regions of small-subunit ribosomal RNA genes. PloS One. 2009;4(7):e6372.

4.	Sisson C, Gulla-Devaney B, Katz LA, Grattepanche JD. Seed bank and seasonal patterns of the eukaryotic SAR (Stramenopila, Alveolata and Rhizaria) clade in a New England vernal pool. J Plankton Res. 2018;40(4):376–90.

5.	Schrecengost A, Rotterová J, Poláková K, Čepička I, Beinart RA. Divergent marine anaerobic ciliates harbor closely related *Methanocorpusculum* endosymbionts. ISME J. 2024;18(1):wrae125.

6.	Kans J. Entrez Direct: E-utilities on the Unix Command Line. In: Entrez Programming Utilities Help. National Center for Biotechnology Information (US); 2025.

7.	Martin M. Cutadapt removes adapter sequences from high-throughput sequencing reads. EMBnet.journal. 2011 May 2;17(1):1.

8.	Bolyen E, Rideout JR, Dillon MR, Bokulich NA, Abnet CC, Al-Ghalith GA, et al. Reproducible, interactive, scalable and extensible microbiome data science using QIIME 2. Nat Biotechnol. 2019 Aug;37(8):8.

9.	Rognes T, Flouri T, Nichols B, Quince C, Mahé F. VSEARCH: a versatile open source tool for metagenomics. PeerJ. 2016 Oct 18;4:e2584.

10.	Amir A, McDonald D, Navas-Molina JA, Kopylova E, Morton JT, Zech Xu Z, et al. Deblur rapidly resolves single-nucleotide community sequence patterns. mSystems. 2(2):e00191-16.

11.	Guillou L, Bachar D, Audic S, Bass D, Berney C, Bittner L, et al. The Protist Ribosomal Reference database (PR2): a catalog of unicellular eukaryote small sub-unit rRNA sequences with curated taxonomy. Nucleic Acids Res. 2012;41(D1):D597–604.

12.	Bokulich NA, Kaehler BD, Rideout JR, Dillon M, Bolyen E, Knight R, et al. Optimizing taxonomic classification of marker-gene amplicon sequences with QIIME 2’s q2-feature-classifier plugin. Microbiome. 2018 May 17;6(1):90.

13.	Nitla V, Serra V, Fokin SI, Modeo L, Verni F, Sandeep BV, et al. Critical revision of the family Plagiopylidae (Ciliophora: Plagiopylea), including the description of two novel species, *Plagiopyla ramani* and *Plagiopyla narasimhamurtii*, and redescription of *Plagiopyla nasuta* Stein, 1860 from India. Zool J Linn Soc. 2019 Apr 22;186(1):1–45.

14.	Li R, Zhuang W, Feng X, Al-Farraj SA, Schrecengost A, Rotterova J, et al. Molecular phylogeny and taxonomy of three anaerobic plagiopyleans (Alveolata: Ciliophora), retrieved from two geographically distant localities in Asia and North America. Zool J Linn Soc. 2023;zlad015.

15.	Katoh K, Rozewicki J, Yamada KD. MAFFT online service: multiple sequence alignment, interactive sequence choice and visualization. Brief Bioinform. 2019 Jul 19;20(4):1160–6.

16.	Larsson A. AliView: a fast and lightweight alignment viewer and editor for large datasets. Bioinformatics. 2014;30(22):3276–8.

17.	Stamatakis A. RAxML version 8: a tool for phylogenetic analysis and post-analysis of large phylogenies. Bioinformatics. 2014;30(9):1312–3.

18.	Czech L, Barbera P, Stamatakis A. Genesis and Gappa: processing, analyzing and visualizing phylogenetic (placement) data. Bioinformatics. 2020 May 15;36(10):3263–5.

19.	Shen W, Le S, Li Y, Hu F. SeqKit: a cross-platform and ultrafast toolkit for FASTA/Q file manipulation. PloS One. 2016;11(10):e0163962.

20.	Evans SN, Matsen FA. The phylogenetic Kantorovich–Rubinstein metric for environmental sequence samples. J R Stat Soc Ser B Stat Methodol. 2012;74(3):569–92.

21.	Matsen IV FA, Evans SN. Edge principal components and squash clustering: using the special structure of phylogenetic placement data for sample comparison. PloS One. 2013;8(3):e56859.

22.	Paradis E, Schliep K. ape 5.0: an environment for modern phylogenetics and evolutionary analyses in R. Bioinformatics. 2019;35(3):526–8.

23.	Camacho C, Coulouris G, Avagyan V, Ma N, Papadopoulos J, Bealer K, et al. BLAST+: architecture and applications. BMC Bioinformatics. 2009 Dec;10(1):421.

24.	Fernandes NM, Vizzoni VF, Borges B do N, Soares CAG, da Silva-Neto ID, Paiva T da S. Molecular phylogeny and comparative morphology indicate that odontostomatids (Alveolata, Ciliophora) form a distinct class-level taxon related to Armophorea. Mol Phylogenet Evol. 2018 Sep;126:382–9.

25.	Wright ES. Using DECIPHER v2. 0 to analyze big biological sequence data in R. 2016.

26.	Oksanen J, Blanchet FG, Friendly M, Kindt R, Legendre P, McGlinn D, et al. vegan: Community Ecology Package. R package version 2.4–3. 2017.

**SUPPLEMENTAL TABLES**

**Table S1.** Studies included in meta-analysis.

**Table S2.** Sample metadata.

**Table S3.** Known free-living anaerobic ciliate taxa.

**Table S4.** Classification of novel ASVs based on patristic distances.

**SUPPLEMENTAL FILES**

File S1. Reference alignment of all sequences used in construction of the APM clade tree for phylogenetic placement.

File S2. Reference 18S rDNA phylogenetic tree of the APM clade used in the phylogenetic placement.

Files S3. Tab-separated file containing information about the tips of the APM clade tree, including GenBank accession IDs and full taxonomic information.

Files S4. Reference alignment of all sequences used in construction of the Plagiopylea clade tree for phylogenetic placement.

File S5. Reference 18S rDNA phylogenetic tree of Plagiopylea used in the phylogenetic placement.

File S6. Tab-separated file containing information about the tips of the Plagiopylea clade tree, including GenBank accession IDs and full taxonomic information.

**SUPPLEMENTAL FIGURES**

**Supplemental Figure 1.** Flowchart describing methodology, from literature review to obtaining anaerobic ciliate sequences. Snakemake pipeline is shown at left in green. User-supplied inputs are represented by dashed-line boxes.

**Supplemental Figure 2.** Phylogenetic placement results of plagiopylid ciliates. Queries which were assigned to Plagiopylea after being placed on the Ciliophora reference tree were then placed onto the Plagiopylea reference tree, which is pictured here. Bootstrap values on reference tree branches are based on 1000 replicates and only values greater than 50 are included. Color on branches indicates log-normalized distribution of top phylogenetic placements for each query, which corresponds to their phylogenetic-placement based taxonomic assignment. Ciliate family is labelled on the right of the tree.

**Supplemental Figure 3.** Phylogenetic placement results of APM ciliates. Queries which were assigned to the APM clade after being placed on the Ciliophora reference tree were then placed onto the APM reference tree, which is pictured here. Color on branches indicates log-normalized distribution of top phylogenetic placements for each query, which corresponds to their phylogenetic-placement based taxonomic assignment. Bootstrap values on reference tree branches are based on 1000 replicates and only values greater than 50 are included. Ciliate family/order/class is labelled on the right of the tree.

**Supplemental Figure 4.** Phylogenetic placement results of Anaerocyclidiidae ciliates. Queries which were assigned to the Anaerocyclidiidae clade after being placed on the Ciliophora reference tree were then placed onto the Anaerocyclidiidae reference tree, which is pictured here. Color on branches indicates log-normalized distribution of top phylogenetic placements for each query, which corresponds to their phylogenetic-placement based taxonomic assignment. Ciliate family/class is labelled on the right of the tree.

 **Supplemental Figure 5. Per-clade summary of ASVs “rescued” by phylogenetic placement.** Left: proportion of “rescued” anaerobic ciliate ASVs per clade which were initially unassigned by QIIME2 and subsequently recovered with our phylogenetic placement pipeline. Right: total number of ASVs assigned to each anaerobic ciliate clade.

**Supplemental Figure 6.** Squash clustering of samples grouped by habitat type. Branch tips are colored by water depth, with deep defined as greater than 300 m, and branch node shapes indicate sample substrate.

**Supplemental Figure 7**. Average patristic distance of ASVs in each anaerobic ciliate clade (A) and habitat type (B). In (A), each ASV was counted once per clade for calculation of the average; in (B) each ASV was counted once per sample (the number of ASVs involved in calculation is shown as *n* at the end of each error bar). Error bars represent standard deviation.

**Supplemental Figure 8. Rarefaction analysis of cumulative ASV discovery** as a function of number of samples for (A) anaerobic ciliate ASVs, (B) Plagiopylea Clade I ASVs, (C) “novel” ASVs (D) and APM clade ASVs. ASVs originated from reads generated from datasets which used the Stoeck et al. 2010 primer set (22 studies, 901 samples). ASVs were clustered at 99% and 98% pairwise identity. Lines represent the mean cumulative ASVs or clusters discovered across 100 random sample orderings and shaded regions indicate the 95% confidence interval.
