# Supplementary material for: A meta-analysis of environmental sequencing data reveals the global distribution and hidden diversity of marine anaerobic ciliates
Source: ISME Commun. 2026 Oct 21;6(1):ycag306. doi: 10.1093/ismeco/ycag306 (PMC13738208; doi:10.1093/ismeco/ycag306)
Supplement: Supplementary file 1 [file supplementary_material_ycag306.zip › FigureS1.pdf]

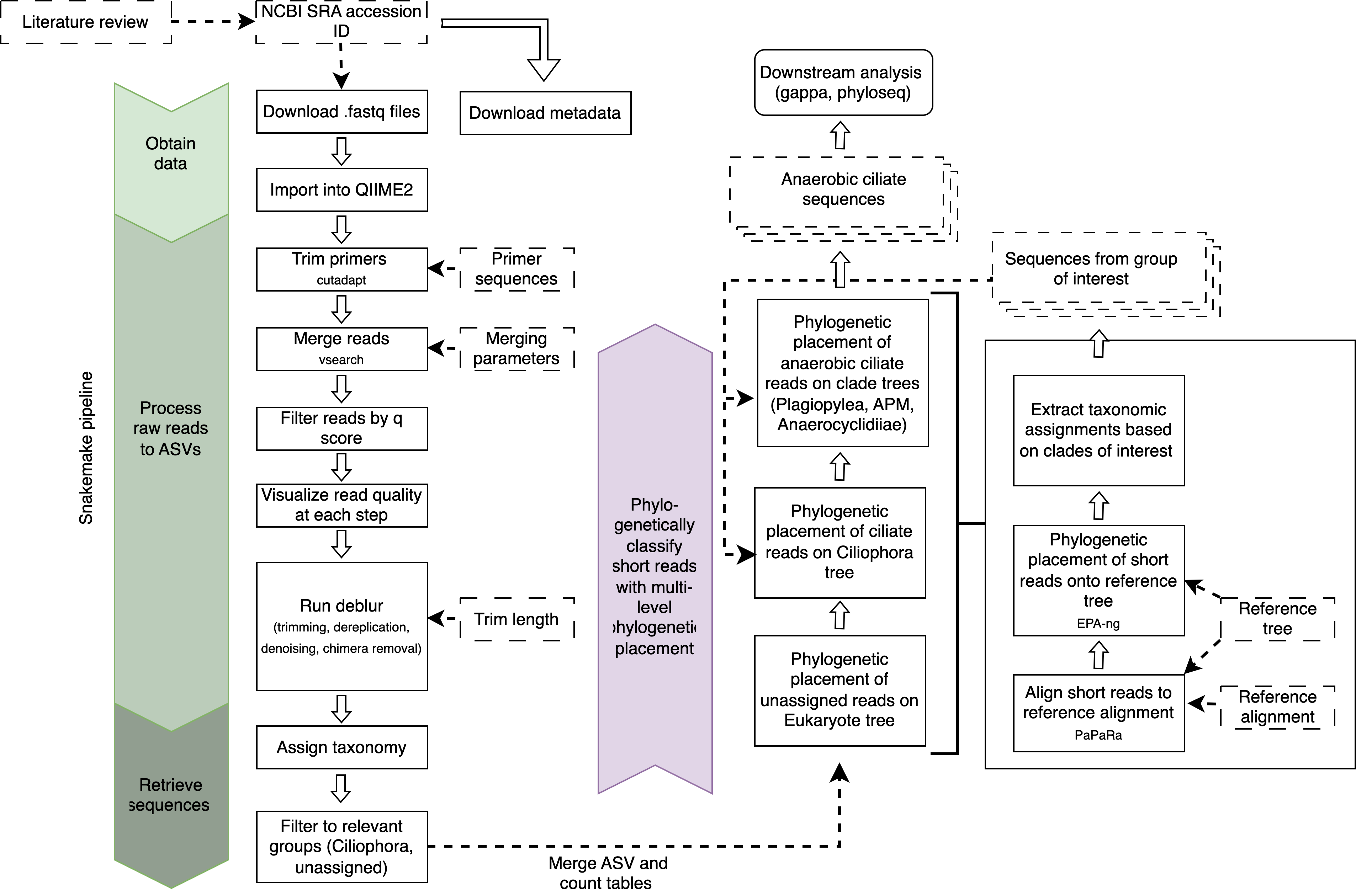
Literature review
NCBI SRA accession ID
Download .fastq files
Download metadata
Obtain data
Import into QIIME2
Trim primers
cutadapt
Primer sequences
Merge reads
vsearch
Merging parameters
Process raw reads to ASVs
Snakemake pipeline
Filter reads by q score
Visualize read quality at each step
Run deblur
(trimming, dereplication, denoising, chimera removal)
Trim length
Assign taxonomy
Retrieve sequences
Filter to relevant groups (Ciliophora, unassigned)
Merge ASV and count tables
Phylo-genetically classify short reads with multi-level phylogenetic placement
Downstream analysis (gappa, phyloseq)
Anaerobic ciliate sequences
Sequences from group of interest
Phylogenetic placement of anaerobic ciliate reads on clade trees (Plagiopylea, APM, Anaerocyclidiiae)
Phylogenetic placement of ciliate reads on Ciliophora tree
Phylogenetic placement of unassigned reads on Eukaryote tree
Extract taxonomic assignments based on clades of interest
Phylogenetic placement of short reads onto reference tree
EPA-ng
Reference tree
Align short reads to reference alignment
PaPaRa
Reference alignment
