# Supplementary material for: A meta-analysis of environmental sequencing data reveals the global distribution and hidden diversity of marine anaerobic ciliates
Source: ISME Commun. 2026 Oct 21;6(1):ycag306. doi: 10.1093/ismeco/ycag306 (PMC13738208; doi:10.1093/ismeco/ycag306)
Supplement: Supplementary file 1 [file supplementary_material_ycag306.zip › FigureS2.pdf]

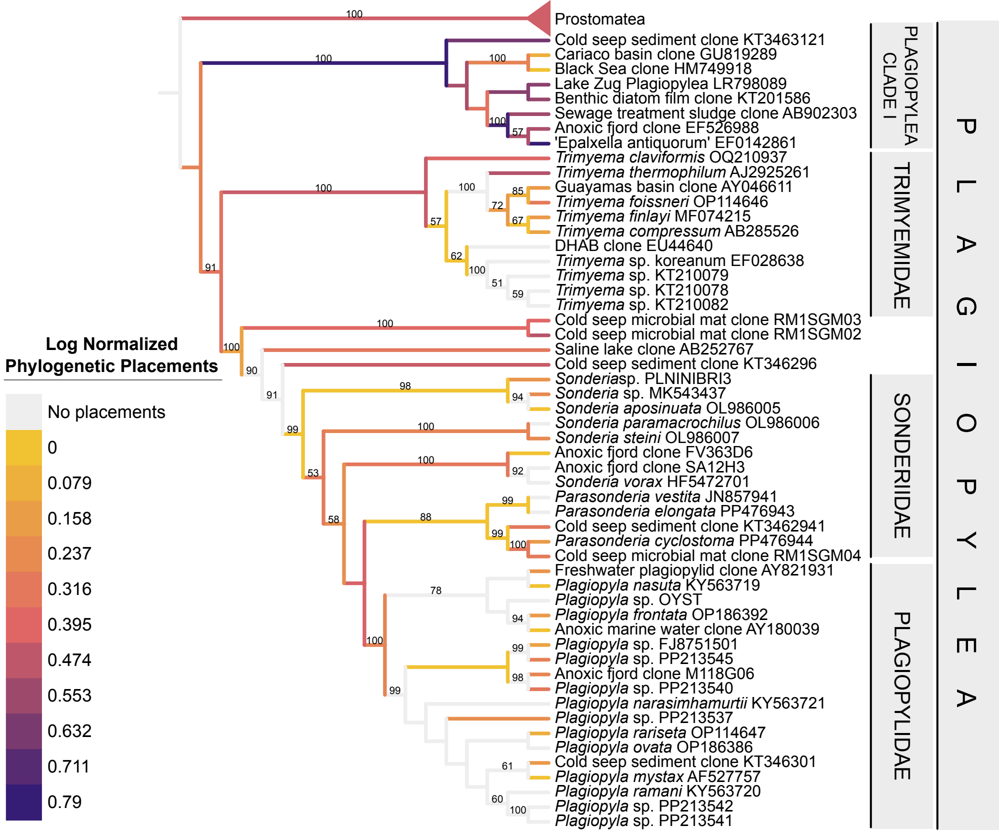

Prostomatea
Cold seep sediment clone KT3463121
Cariaco basin clone GU819289
Black Sea clone HM749918
Lake Zug Plagiopylea LR798089
Benthic diatom film clone KT201586
Sewage treatment sludge clone AB902303
Anoxic fjord clone EF526988
'Epalxella antiquorum' EF0142861
Trimyema claviformis OQ210937
Trimyema thermophilum AJ2925261
Guayamas basin clone AY046611
Trimyema foissneri OP114646
Trimyema finlayi MF074215
Trimyema compressum AB285526
DHAB clone EU44640
Trimyema sp. koreanum EF028638
Trimyema sp. KT210079
Trimyema sp. KT210078
Trimyema sp. KT210082
Cold seep microbial mat clone RM1SGM03
Cold seep microbial mat clone RM1SGM02
Saline lake clone AB252767
Cold seep sediment clone KT346296
Sonderiasp. PLNINIBRI3
Sonderia sp. MK543437
Sonderia aposinuata OL986005
Sonderia paramacrochilus OL986006
Sonderia steini OL986007
Anoxic fjord clone FV363D6
Anoxic fjord clone SA12H3
Sonderia vorax HF5472701
Parasonderia vestita JN857941
Parasonderia elongata PP476943
Cold seep sediment clone KT3462941
Parasonderia cyclostoma PP476944
Cold seep microbial mat clone RM1SGM04
Freshwater plagiopylid clone AY821931
Plagiopyla nasuta KY563719
Plagiopyla sp. OYST
Plagiopyla frontata OP186392
Anoxic marine water clone AY180039
Plagiopyla sp. FJ8751501
Plagiopyla sp. PP213545
Anoxic fjord clone M118G06
Plagiopyla sp. PP213540
Plagiopyla narasimhamurtii KY563721
Plagiopyla sp. PP213537
Plagiopyla rariseta OP114647
Plagiopyla ovata OP186386
Cold seep sediment clone KT346301
Plagiopyla mystax AF527757
Plagiopyla ramani KY563720
Plagiopyla sp. PP213542
Plagiopyla sp. PP213541
PLAGIOPYLEA CLADE I
TRIMYEMIDAE
SONDERIDAE
PLAGIOPYLIDAE
PLAGIOPYLEA
Log Normalized Phylogenetic Placements
No placements
0
0.079
0.158
0.237
0.316
0.395
0.474
0.553
0.632
0.711
0.79
