# Supplementary material for: A meta-analysis of environmental sequencing data reveals the global distribution and hidden diversity of marine anaerobic ciliates
Source: ISME Commun. 2026 Oct 21;6(1):ycag306. doi: 10.1093/ismeco/ycag306 (PMC13738208; doi:10.1093/ismeco/ycag306)
Supplement: Supplementary file 1 [file supplementary_material_ycag306.zip › FigureS3.pdf]

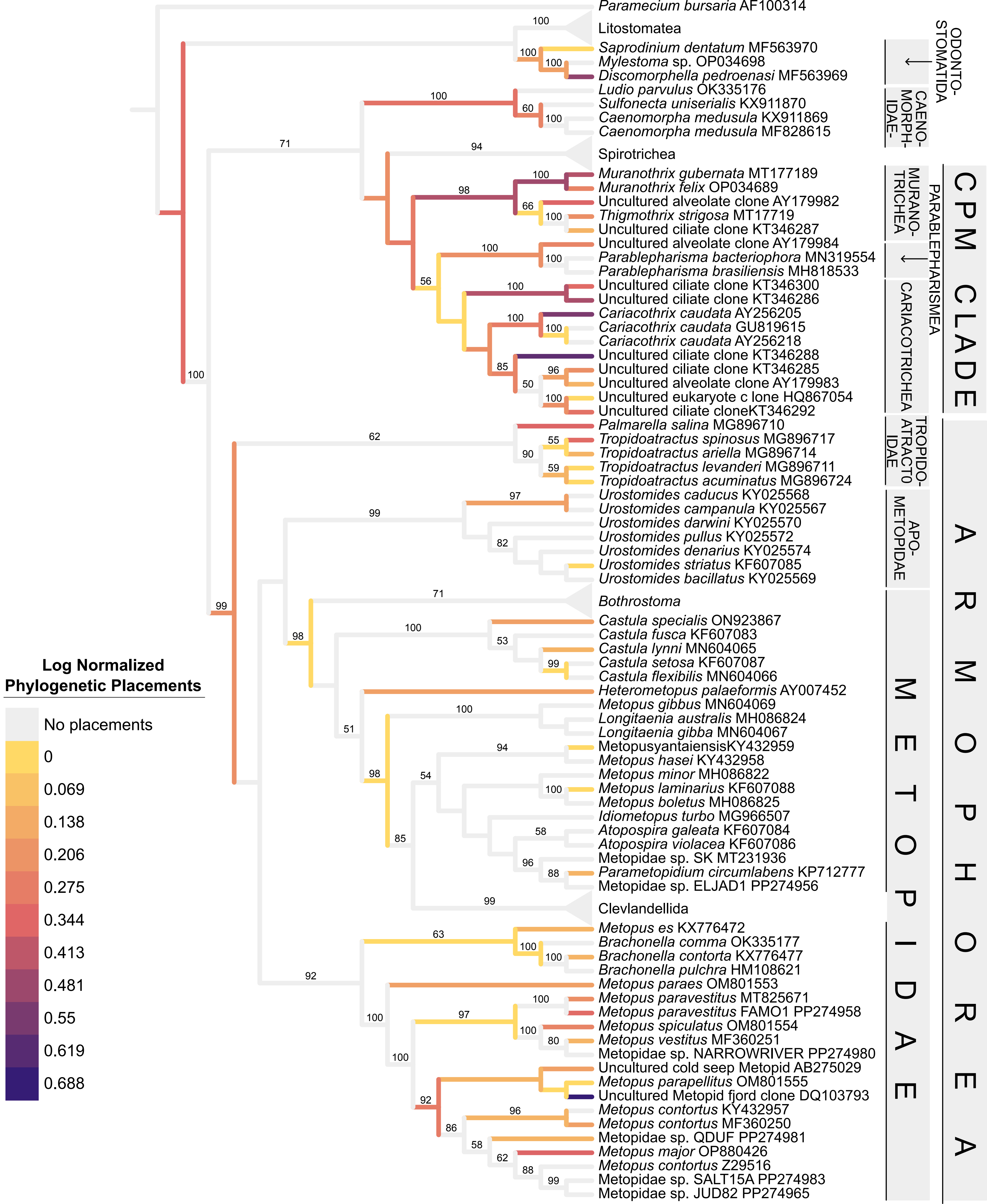

Paramecium bursaria AF100314
Litostomatea
Saprodinium dentatum MF563970
Mylestoma sp. OP034698
Discomorphella pedroenasi MF563969
Ludio parvulus OK335176
Sulfonecta uniserialis KX911870
Caenomorpha medusula KX911869
Caenomorpha medusula MF828615
Spirotrichea
Muranothrix gubernata MT177189
Muranothrix felix OP034689
Uncultured alveolate clone AY179982
Thigmothrix strigosa MT17719
Uncultured ciliate clone KT346287
Uncultured alveolate clone AY179984
Parablepharisma bacteriophora MN319554
Parablepharisma brasiliensis MH818533
Uncultured ciliate clone KT346300
Uncultured ciliate clone KT346286
Cariacothrix caudata AY256205
Cariacothrix caudata GU819615
Cariacothrix caudata AY256218
Uncultured ciliate clone KT346288
Uncultured ciliate clone KT346285
Uncultured alveolate clone AY179983
Uncultured eukaryote c lone HQ867054
Uncultured ciliate cloneKT346292
Palmarella salina MG896710
Tropidoatractus spinosus MG896717
Tropidoatractus ariella MG896714
Tropidoatractus levanderi MG896711
Tropidoatractus acuminatus MG896724
Urostomides caducus KY025568
Urostomides campanula KY025567
Urostomides darwini KY025570
Urostomides pullus KY025572
Urostomides denarius KY025574
Urostomides striatus KF607085
Urostomides bacillatus KY025569
Bothrostoma
Castula specialis ON923867
Castula fusca KF607083
Castula lynni MN604065
Castula setosa KF607087
Castula flexibilis MN604066
Heterometopus palaeformis AY007452
Metopus gibbus MN604069
Longitaenia australis MH086824
Longitaenia gibba MN604067
MetopusyantaiensisKY432959
Metopus hasei KY432958
Metopus minor MH086822
Metopus laminarius KF607088
Metopus boletus MH086825
Idiometopus turbo MG966507
Atopospira galeata KF607084
Atopospira violacea KF607086
Metopidae sp. SK MT231936
Parametopidium circumlabens KP712777
Metopidae sp. ELJAD1 PP274956
Clevlandellida
Metopus es KX776472
Brachonella comma OK335177
Brachonella contorta KX776477
Brachonella pulchra HM108621
Metopus paraes OM801553
Metopus paravestitus MT825671
Metopus paravestitus FAMO1 PP274958
Metopus spiculatus OM801554
Metopus vestitus MF360251
Metopidae sp. NARROWRIVER PP274980
Uncultured cold seep Metopid AB275029
Metopus parapellitus OM801555
Uncultured Metopid fjord clone DQ103793
Metopus contortus KY432957
Metopus contortus MF360250
Metopidae sp. QDUF PP274981
Metopus major OP880426
Metopus contortus Z29516
Metopidae sp. SALT15A PP274983
Metopidae sp. JUD82 PP274965
ODONTO-STOMATIDA
CAENO-MORPH-IDAE-
MURANO-TRICHEA
PARABLEPHARISMEA
CARIACOTRICHEA
CPM CLADE
TROPIDO-ATRACTO IDAE
APO-METOPIDAE
METOPIDAE
ARMOPHOREA
Log Normalized Phylogenetic Placements
No placements
0
0.069
0.138
0.206
0.275
0.344
0.413
0.481
0.55
0.619
0.688
