# Supplementary material for: A meta-analysis of environmental sequencing data reveals the global distribution and hidden diversity of marine anaerobic ciliates
Source: ISME Commun. 2026 Oct 21;6(1):ycag306. doi: 10.1093/ismeco/ycag306 (PMC13738208; doi:10.1093/ismeco/ycag306)
Supplement: Supplementary file 1 [file supplementary_material_ycag306.zip › FigureS4.pdf]

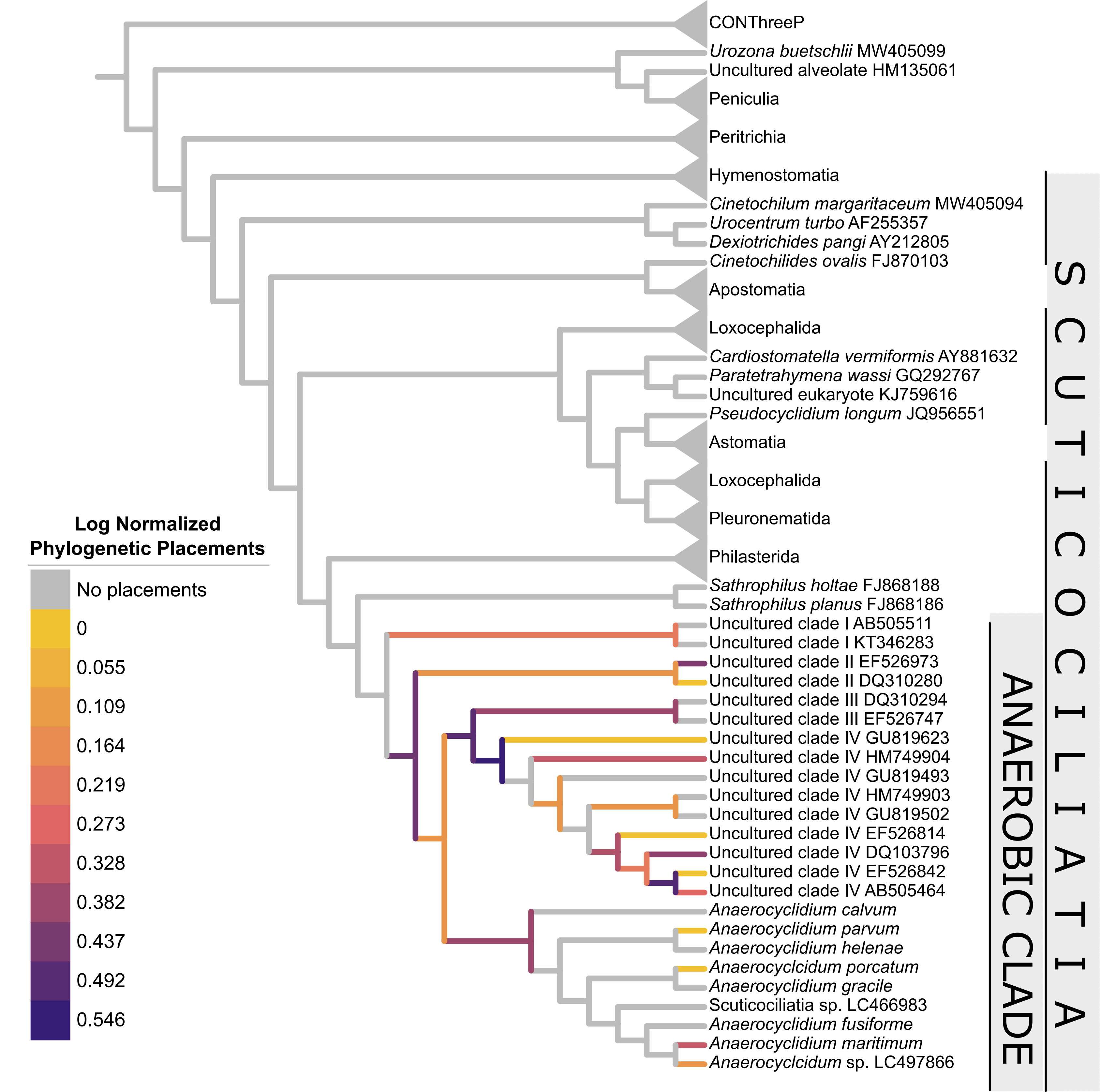

CONThreeP
Urozona buetschlii MW405099
Uncultured alveolate HM135061
Peniculia
Peritrichia
Hymenostomatia
Cinetochilum margaritaceum MW405094
Urocentrum turbo AF255357
Dexiotrichides pangi AY212805
Cinetochilides ovalis FJ870103
Apostomatia
Loxocephalida
Cardiostomatella vermiformis AY881632
Paratetrahymena wassi GQ292767
Uncultured eukaryote KJ759616
Pseudocyclidium longum JQ956551
Astomatia
Loxocephalida
Pleuronematida
Philasterida
Sathrophilus holtae FJ868188
Sathrophilus planus FJ868186
Uncultured clade I AB505511
Uncultured clade I KT346283
Uncultured clade II EF526973
Uncultured clade II DQ310280
Uncultured clade III DQ310294
Uncultured clade III EF526747
Uncultured clade IV GU819623
Uncultured clade IV HM749904
Uncultured clade IV GU819493
Uncultured clade IV HM749903
Uncultured clade IV GU819502
Uncultured clade IV EF526814
Uncultured clade IV DQ103796
Uncultured clade IV EF526842
Uncultured clade IV AB505464
Anaerocyclidium calvum
Anaerocyclidium parvum
Anaerocyclidium helenae
Anaerocyclcidum porcatum
Anaerocyclidium gracile
Scuticociliatia sp. LC466983
Anaerocyclidium fusiforme
Anaerocyclidium maritimum
Anaerocyclcidum sp. LC497866
SCUTICOCILIATIA
ANAEROBIC CLADE
Log Normalized Phylogenetic Placements
No placements
0
0.055
0.109
0.164
0.219
0.273
0.328
0.382
0.437
0.492
0.546
