# Supplementary material for: A meta-analysis of environmental sequencing data reveals the global distribution and hidden diversity of marine anaerobic ciliates
Source: ISME Commun. 2026 Oct 21;6(1):ycag306. doi: 10.1093/ismeco/ycag306 (PMC13738208; doi:10.1093/ismeco/ycag306)
Supplement: Supplementary file 1 [file supplementary_material_ycag306.zip › FigureS5.pdf]

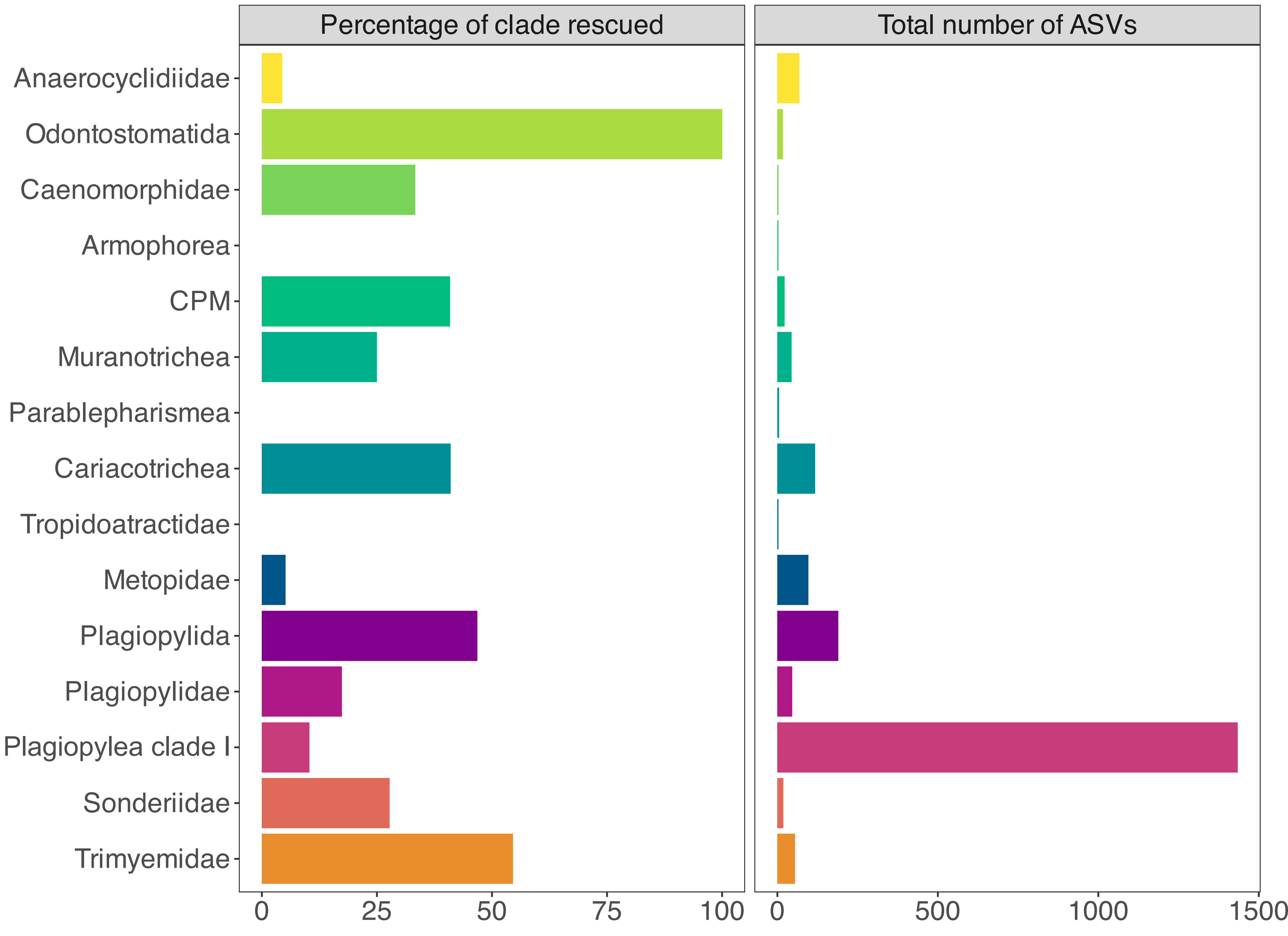

Percentage of clade rescued
Total number of ASVs
Anaerocyclidiidae
Odontostomatida
Caenomorphidae
Armophorea
CPM
Muranotrichea
Parablepharismea
Cariacotrichea
Tropidoatractidae
Metopidae
Plagiopylida
Plagiopylidae
Plagiopylea clade I
Sonderiidae
Trimyemidae
0
25
50
75
100
0
500
1000
1500
