# Supplementary material for: A meta-analysis of environmental sequencing data reveals the global distribution and hidden diversity of marine anaerobic ciliates
Source: ISME Commun. 2026 Oct 21;6(1):ycag306. doi: 10.1093/ismeco/ycag306 (PMC13738208; doi:10.1093/ismeco/ycag306)
Supplement: Supplementary file 1 [file supplementary_material_ycag306.zip › FigureS6.pdf]

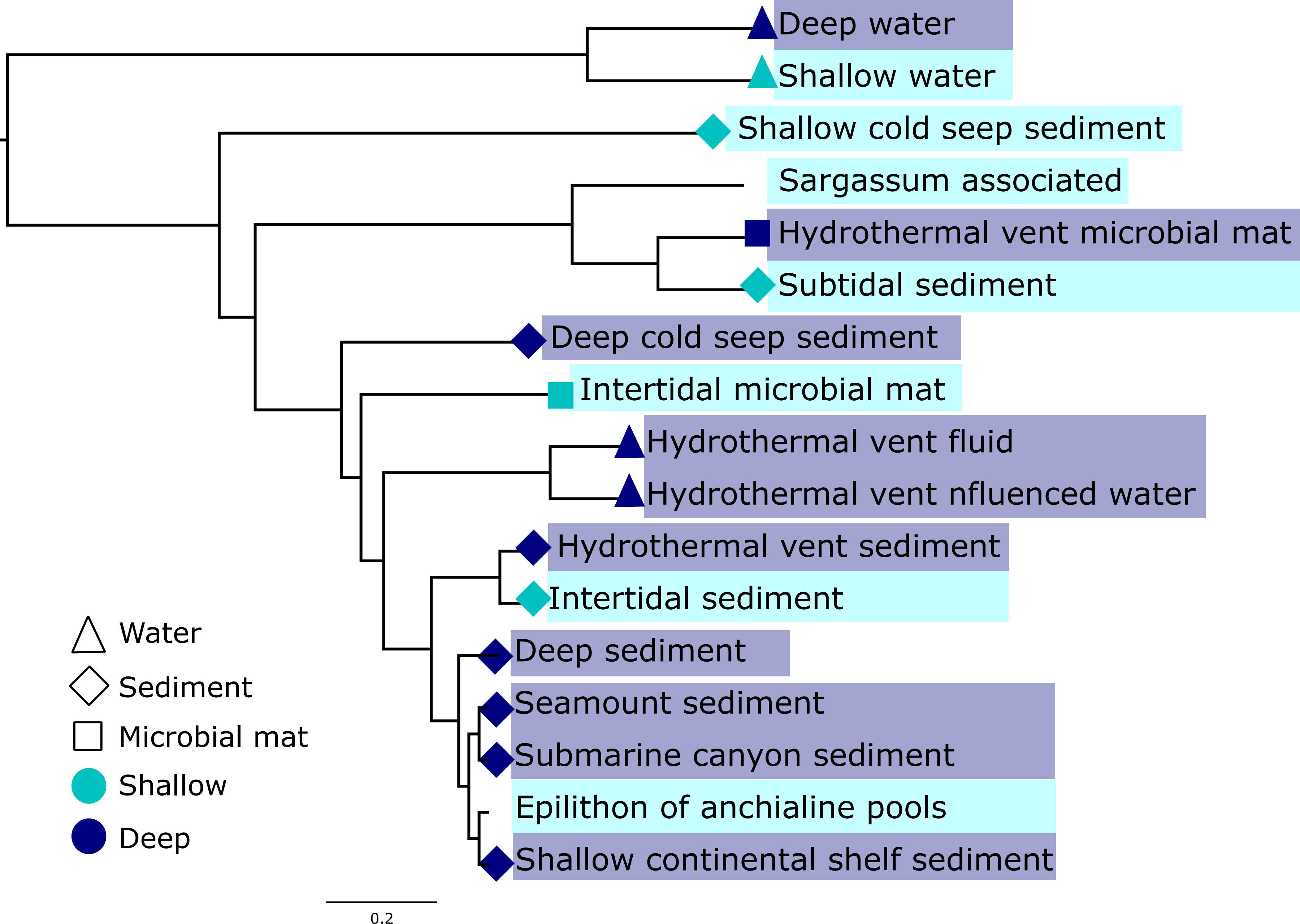
Deep water
Shallow water
Shallow cold seep sediment
Sargassum associated
Hydrothermal vent microbial mat
Subtidal sediment
Deep cold seep sediment
Intertidal microbial mat
Hydrothermal vent fluid
Hydrothermal vent nfluenced water
Hydrothermal vent sediment
Intertidal sediment
Deep sediment
Seamount sediment
Submarine canyon sediment
Epilithon of anchialine pools
Shallow continental shelf sediment
Water
Sediment
Microbial mat
Shallow
Deep
0.2
