# Supplementary material for: A meta-analysis of environmental sequencing data reveals the global distribution and hidden diversity of marine anaerobic ciliates
Source: ISME Commun. 2026 Oct 21;6(1):ycag306. doi: 10.1093/ismeco/ycag306 (PMC13738208; doi:10.1093/ismeco/ycag306)
Supplement: Supplementary file 1 [file supplementary_material_ycag306.zip › FigureS7.pdf]

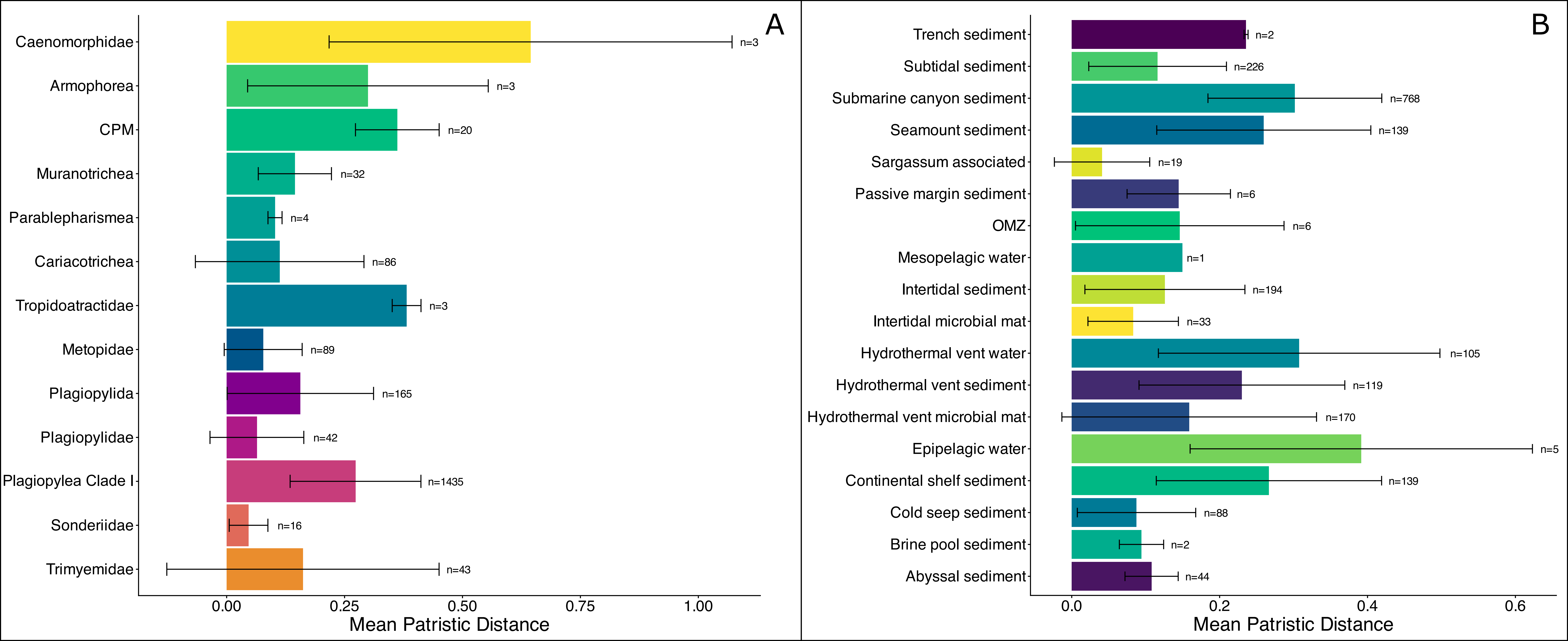
A
Caenomorphidae
Armophorea
CPM
Muranotrichea
Parablepharismea
Cariacotrichea
Tropidoatractidae
Metopidae
Plagiopylida
Plagiopylidae
Plagiopylea Clade I
Sonderiidae
Trimyemidae
n=3
n=3
n=20
n=32
n=4
n=86
n=3
n=89
n=165
n=42
n=1435
n=16
n=43
0.00
0.25
0.50
0.75
1.00
Mean Patristic Distance
B
Trench sediment
Subtidal sediment
Submarine canyon sediment
Seamount sediment
Sargassum associated
Passive margin sediment
OMZ
Mesopelagic water
Intertidal sediment
Intertidal microbial mat
Hydrothermal vent water
Hydrothermal vent sediment
Hydrothermal vent microbial mat
Epipelagic water
Continental shelf sediment
Cold seep sediment
Brine pool sediment
Abyssal sediment
n=2
n=226
n=768
n=139
n=19
n=6
n=6
n=1
n=194
n=33
n=105
n=119
n=170
n=5
n=139
n=88
n=2
n=44
0.0
0.2
0.4
0.6
Mean Patristic Distance
