# Supplementary material for: A meta-analysis of environmental sequencing data reveals the global distribution and hidden diversity of marine anaerobic ciliates
Source: ISME Commun. 2026 Oct 21;6(1):ycag306. doi: 10.1093/ismeco/ycag306 (PMC13738208; doi:10.1093/ismeco/ycag306)
Supplement: Supplementary file 1 [file supplementary_material_ycag306.zip › FigureS8.pdf]

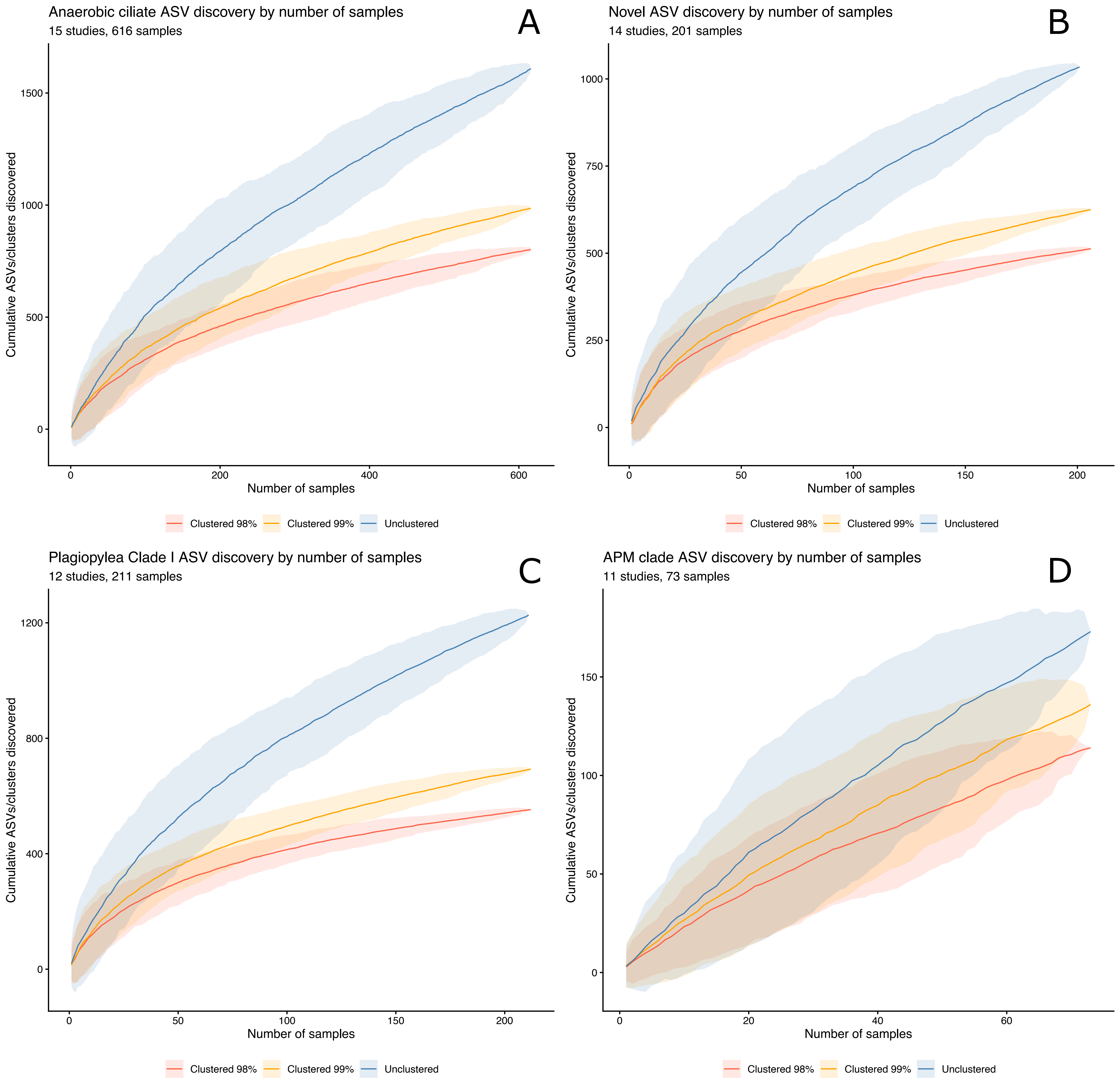
Anaerobic ciliate ASV discovery by number of samples
15 studies, 616 samples
A
Cumulative ASVs/clusters discovered
Number of samples
Clustered 98%
Clustered 99%
Unclustered
Novel ASV discovery by number of samples
14 studies, 201 samples
B
Plagiopylea Clade I ASV discovery by number of samples
12 studies, 211 samples
C
APM clade ASV discovery by number of samples
11 studies, 73 samples
D
